# Precancerous cervical lesions and associated factors among HIV-positive women in Southern and Eastern African countries: A systematic review and meta-analysis

**DOI:** 10.1371/journal.pone.0361185

**Published:** 2026-10-23

**Authors:** Gebrie Getu Alemu, Astewil Moges Bazezew, Amlaku Nigusie Yirsaw, Wubet Tazeb Wondie, Berihun Agegn Mengistie, Tenagnework Eseyneh Dagnaw, Mekuriaw Nibret Aweke, Nebebe Demis Baykemagn, Gebeyehu Lakew, Bisrat Tewelde Gebretsadkan

**Affiliations:** 1 Department of Epidemiology and Biostatistics, Institute of Public Health, College of Medicine and Health Sciences, University of Gondar, Gondar, Ethiopia; 2 Department of Surgical Nursing, School of Nursing, College of Medicine and Health Sciences, University of Gondar, Gondar, Ethiopia; 3 Department of Health Promotion and Health Behavior, Institute of Public Health, College of Medicine and Health Sciences, University of Gondar, Gondar, Ethiopia; 4 Department of Pediatrics and Child Health Nursing, College of Medicine and Health Sciences, Bahir Dar University, Bahir Dar, Ethiopia; 5 Department of General Midwifery, School of Midwifery, College of Medicine and Health Sciences, University of Gondar, Gondar, Ethiopia; 6 Department of Public Health, College of Medicine and Health Sciences, Injibara University, Injibara, Ethiopia; 7 Department of Human Nutrition, Institute of Public Health, College of Medicine and Health Sciences, University of Gondar, Gondar, Ethiopia; 8 Department of Health Informatics, Institute of Public Health, College of Medicine and Health Sciences, University of Gondar, Gondar, Ethiopia; 9 Department of Environmental Health and Behavioral Sciences, School of Public Health, College of Medicine and Health Sciences, Mekelle University, Mekelle, Ethiopia; UFRN: Universidade Federal do Rio Grande do Norte, BRAZIL

## Abstract

**Background:**

Women living with HIV (WLHIV) face a sixfold increased risk of cervical cancer compared to HIV-negative women. Most WLHIV residing in the Southern and Eastern African regions have a substantial HIV-related cervical cancer burden. Studies on precancerous cervical lesions (PCCL) and associated factors in WLHIV from these regions have inconsistent and inconclusive findings. Therefore, we aimed to synthesize the pooled prevalence of PCCL and associated factors among WLHIV in Southern and Eastern Africa.

**Methods:**

This study was registered under PROSPERO (CRD420251123583) and conducted in accordance with PRISMA 2020 standards. Primary studies published from inception to 2025 in Southern and Eastern Africa were included. The methodological quality of studies was assessed using the Newcastle-Ottawa scale. Data extraction and statistical analyses were done in Microsoft Excel and STATA 17, respectively. Pooled prevalence, publication bias, subgroup, and sensitivity analysis were conducted. Associations were expressed as odds ratios (OR), and a p-value of less than 0.05 was considered statistically significant.

**Results:**

44 studies met the inclusion criteria, with a sample size of 47,009. The pooled prevalence of PCCL was 22.25% (95% CI: 19.57, 24.93). Age at first sexual intercourse (< 18 years) (OR = 2.99, (2.34, 3.82)), multiparity (OR = 4.97, (3.21, 7.69)), having multiple sexual partners (OR = 3.06 (2.45, 3.83)), having a history of sexually transmitted infections (STI) (OR = 3.93 (3.23, 4.78)), smoking (OR = 7.01, (3.39, 14.49)), ever taking oral contraceptive pills (OR = 2.69, (1.85, 3.90)), low CD4 count (OR = 1.79, (1.56, 2.05)), and having not initiated antiretroviral therapy (ART) (OR = 2.02, (1.56, 2.62)) were significantly associated with an increased risk of PCCL.

**Conclusion and recommendations:**

The pooled prevalence of PCCL was high. Additionally, early first sexual intercourse, parity, STIs, multiple sexual partners, smoking, oral contraceptive use, CD4 count, and ART were positively associated with the occurrence of PCCL. Therefore, stakeholders shall implement interventions to avert future incidents of PCCL and address the identified factors.

## Introduction

A precancerous cervical lesion (PCCL) is a cervical intraepithelial neoplasia or squamous intraepithelial lesion that can progress to cervical cancer in people living with human immunodeficiency virus (HIV) if untreated [1]. Cervical cancer is largely preventable through human papillomavirus (HPV) vaccination and regular screening and is curable when detected and treated early [2].

Cervical cancer screening is intended to identify women who are at risk and to make sure that those who have a positive or abnormal test result receive the proper follow-up. The World Health Organization (WHO) advises using a “screen-and-treat” strategy to detect and cure precancerous lesions in order to avoid cervical cancer. Following a positive screening test, treatment should be administered quickly. Screening tests include Human Papillomavirus Deoxyribonucleic Acid (HPV DNA), Visual Inspection with Acetic Acid (VIA) or Visual Inspection with Lugol's Iodine (VILI), and the Pap smear/cytology [3,4].

The WHO has set new ambitious targets to eliminate cervical cancer [2,5]. Besides the goal, it was insufficient to eliminate cervical cancer among WLHIV [6], and a study predicts that new cases are expected to rise by 2050 [5]. Globally, 99.7% of cervical precancerous and malignant lesions are attributable to HPV [7]. HIV-positive women are more likely to develop PCCL and invasive cervical cancer. They are 6 times more likely to develop cervical cancer compared to women without HIV [2].

Cervical cancer incidence and mortality are highest in low- and middle-income countries (LMICs), largely due to lack of access to HPV vaccination, screening, and socioeconomic determinants [2]. Approximately 85% of WLHIV and cervical cancer cases occur in sub-Saharan Africa (SSA), highlighting the region’s high cervical cancer burden and persistent inequalities [5]. The most affected regions with the double burden of diseases were Southern and Eastern Africa (SEA). In 2018, HIV prevalence among women with cervical cancer was 63.8% in Southern Africa and 27.4% in Eastern Africa [8].

Understanding the factors associated with PCCL is critical to create evidence-based interventions to prevent cervical cancer [9]. Socioeconomic and demographic factors, healthcare access, and behavioral and clinical factors, including poverty, age, education, parity, distances to services, sexual behavior, absence of screening facilities, CD4 count, viral load, STIs, ART use, oral contraceptives, and tobacco use, are listed among the contributing factors for the occurrence of PCCL [10–25].

There have been conflicting results and conclusions from studies that assessed the prevalence and associated factors of the PCCL among WLHIV across SEA countries, ranging from 1.87% in Zimbabwe [26] to 76.00% in Zambia [27]. Small, independent studies may yield inconclusive results, but combining data increases precision. Therefore, the objective of this systematic review and meta-analysis (SRMA) was to determine the pooled prevalence of PCCL and identify associated factors. This study will give insight to policymakers, program planners, researchers, clinicians, and other key stakeholders to implement an intervention that prevents cervical cancer and will provide better guidance for future interventions.

## Methods and materials

This SRMA was conducted in accordance with the Preferred Reporting Items for Systematic Reviews and Meta-Analyses (PRISMA 2020) guidelines [28] (S1 Table). The protocol was registered in the International Prospective Register of Systematic Reviews (PROSPERO) database at (https://www.crd.york.ac.uk/PROSPERO/view/CRD420251123583).

### Eligibility criteria

#### Inclusion criteria.

We included original primary studies, both published and unpublished, in any language reporting data on the PCCL and/or associated factors among WLHIV in Southern and Eastern Africa, from inception through August 15, 2025.

#### Exclusion criteria.

Studies that did not report the outcomes of interests, reviews, case studies, letters, protocols, studies without full texts [29,30], and articles that failed to satisfy the quality assessment standards were excluded [31,32].

### Information sources and search strategy

An inclusive literature search for primary studies on PCCL and associated factors was conducted using the Condition, Context, and Population (CoCoPop) framework across electronic databases, including PubMed/MEDLINE, Embase, Scopus, Google Scholar, ScienceDirect, and Hinari, to retrieve potential studies relevant to the objective of this study. Additionally, the snowballing technique was conducted. Furthermore, manual searches were performed across different repositories to identify grey literature. Search strategies were adapted for each selected database using relevant search terms and combinations of terms in a systematic search syntax using Boolean operators (“AND” and “OR). The literature search was conducted from July 25 to August 15, 2025, and updated in August 2026. The following search syntaxes and phrases were used for searching. Precancerous; “precancerous cervical lesion”; “cervical lesion”; “Cervical Intraepithelial Neoplasia”; “Cervical cancer”; “HIV-positive women”; “HIV Infections”; “HIV-Sero positivity”; “Women living with HIV”; Determinants; Predictors; “Associated Factors”; “Risk Factors”; Barriers; Burundi; Botswana; Comoros; Djibouti; Eritrea; Eswatini; Ethiopia; Kenya; Lesotho; Madagascar; Malawi; Mauritius; Mozambique; Namibia; Rwanda; Seychelles; Somalia; “South Africa”; “South Sudan”; Swaziland; Tanzania; Uganda; Zambia; Zimbabwe; “Eastern Africa”; “Southern Africa.” The details of the electronic data bases, the search strategy, and the corresponding numbers are supplied (S2 Table).

### Selection process

All retrieved records were exported into EndNote 20 to remove duplicates and then evaluated for eligibility. The retrieved data were screened independently by two authors (GGA and MNA) according to the review's eligibility criteria. The evaluation process was conducted at three stages: title, abstract, and full-text. The full texts of potentially eligible abstracts were downloaded and examined to verify whether they fulfilled the inclusion criteria. Any disagreement was resolved through discussion with a third reviewer (AMB).

### Data extraction

Data extraction was carried out separately by two reviewers (GGA and NDB) utilizing a prearranged Microsoft Excel. The extracted data items include the first author's name, year of publication, study country, sample size, response rate, and variables related to PCCL and OR with 95% CIs.

### Study quality and risk of bias assessment

The quality of the included studies was independently assessed by three authors (GGA, BTG, and MNA). Disagreements in the assessment and scoring were solved through discussion and consensus. The quality of studies was assessed using the Newcastle‒Ottawa Scale (NOS). The tool was adapted for the purpose of this study. It included 3 categorical criteria comprising selection (maximum 5 scores), comparability (maximum 2 scores), and outcome (maximum 3 scores) with a total maximum score of 10 points. The quality of the included studies was rated according to the following scale: Very good studies: 9–10 points; good studies: 7–8 points; satisfactory studies: 5–6 points; unsatisfactory studies: 0–4 points. Studies with satisfactory to very good quality were included (S3 Table) [33,34].

### Effect measures

The primary outcome of interest for this review was the pooled prevalence of PCCL, estimated as the total number of WLHIV with lesions divided by the total number of WLHIV taking part in the study multiplied by 100%. The secondary outcomes were factors associated with PCCL. We assessed the association between precancerous lesions of the cervix and its determinants in the form of the log odds ratio. For determinants, the odds ratio was calculated based on binary outcomes from the primary studies.

### Data synthesis and analysis

The extracted data were imported into STATA 17 software for comprehensive analysis. The analyzed data were presented as texts, tables, and figures. The margin of error for the prevalence in each study was calculated using a binomial probability model. The combined prevalence from the studies was evaluated for heterogeneity using the I^2^ statistic. The proportions of 25%, 50%, and 75% were defined as low, moderate, and high heterogeneity, respectively [35]. We performed a meta-analysis to estimate the pooled prevalence of PCCL. Subgroup analyses were conducted for geographical area, year of publication, and screening methods. Similarly, a leave-one-out sensitivity analysis was performed to evaluate the robustness of the synthesized results. Publication bias was assessed visually by funnel plots and quantitatively by the Egger regression model. For the variables associated with PCCL, the OR along with its 95% CIs and p < 0.05 was used as the significance threshold.

### Operational definitions

**Cervical intraepithelial neoplasia:** a histological diagnosis made upon analysis of cervical tissue obtained by biopsy or surgical excision. The condition is graded as CIN 1, 2, or 3, according to the thickness of the abnormal epithelium (1/3, 2/3, or the entire thickness).

**Squamous intraepithelial lesion:** an abnormal result derived from cervical cytological screening or Pap smear testing.

**Low-grade squamous intraepithelial lesions (LSIL/CIN-1)** are defined by early changes in size, shape, and number of abnormal cells formed on the surface of the cervix and may be referred to as mild dysplasia, LSIL, or CIN-1.

**High-grade squamous intraepithelial lesions (HSIL/CIN-2/CIN-3/CIS)** are defined by a large number of precancerous cells on the surface of the cervix that are distinctly different from normal cells. They have the potential to become cancerous cells and invade deeper tissues of the cervix. These lesions may be referred to as moderate or severe dysplasia, HSIL, CIN-2, CIN-3, or cervical carcinoma in situ (CIS).

**Carcinoma in situ (CIS):** pervasive malignancy limited to the epithelium without invasion of the basement membrane.

**Invasive cervical cancer:** a malignant tumor that has spread from the cervix to the tissue deeper in the cervix or to other parts of the body. Invasive cervical cancer stages range from stage I (cancer is in the cervix or uterus only) to stage IV (the cancer has spread to distant organs, such as the liver).

### Ethical statements

The study utilized publicly available primary studies. No new original data involving human participants was gathered. Consequently, ethical approval was not necessary for this study.

## Results

### Identification and selection of the studies

A total of 2070 studies were identified through database searches and from manual sources. After removing 542 duplicates, 1528 articles were screened by title and abstract; 1433 were subsequently excluded due to being unrelated to the title and abstract contents. Then 95 full-text articles were assessed for eligibility, but 51 were excluded with reason through the critical review process (S4 Table). Finally, 44 articles met the inclusion criteria for determining the pooled prevalence of PCCL and its associated factors and included (Fig 1).

**Fig 1:**
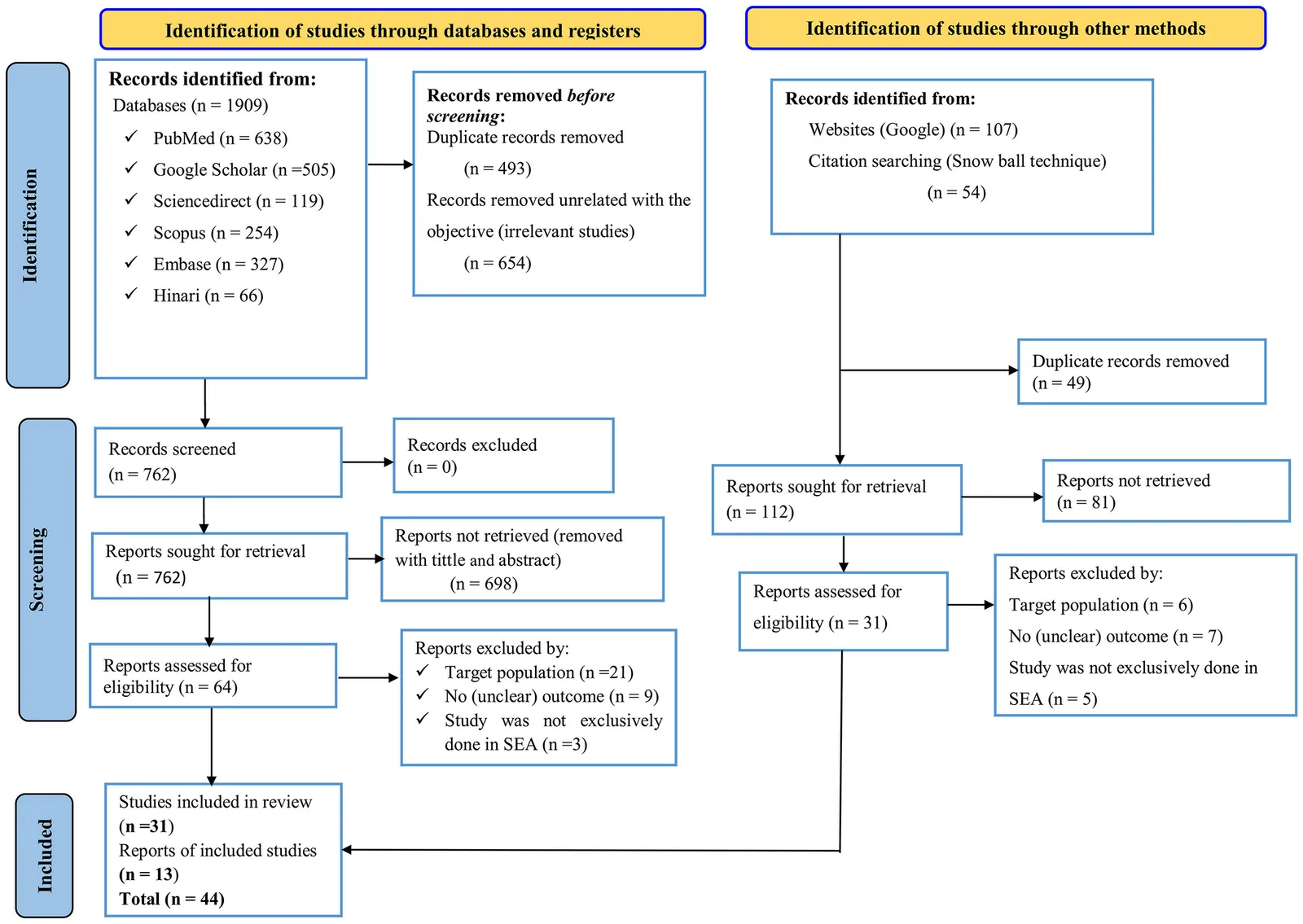
A PRISMA flow diagram illustrating study selection for the pooled prevalence of PCCL and its associated factors among WLHIV in Southern and Eastern Africa, 2025.

### Study characteristics

This study included 44 articles involving a total of 47,009 participants, with the sample size and response rate ranging from 49 to 16,869 and 84.05% to 100%, respectively. Regarding the distribution of studies, fifteen were conducted in Ethiopia [11,13–15,21,22,25,36–43], ten in Kenya [1,19,44–51], four each in Uganda [17,52–54] and Tanzania [12,23,55,56], three in South Africa [24,57,58], two each in Namibia [59,60] and Zambia [27,61], and one each in Rwanda [62], Botswana [63], Eswatinia [18], and Zimbabwe [26]. Moreover, 33 studies were conducted using a cross-sectional design, 4 using a cohort design, 2 using a case-control design, 1 using a randomized control trial, and the remaining 4 were not reported on study design. Regarding the screening methods, 26 were conducted using VIA/VILI, 15 using Pap smear/cytology, and 3 using HPV DNA (Table 1).

**Table 1:** Descriptive characteristics of the included studies for premalignant cervical lesions and associated factors, 2025.

No	Author, Year	Study country	Screening methods	Study design	Response rate (%)	Sample size	Prevalence (%)	Quality (NOS)	Risk of bias
1	Bateman AC, 2015 [61]	Zambia	VIA	CS	100	309	52.10	7	Low
2	Belayneh T, 2019 [11]	Ethiopia	Pap smear/cytology	CS	97.6	284	9.86	8	Low
3	Chambuso RS, 2017 [12]	Tanzania	VIA/VILI	CS	100	110	71.82	7	Low
4	Dessie TM, 2023 [13]	Ethiopia	VIA	CC	100	312	NA	9	Very low
5	Dinberia AH 2025 [36]	Ethiopia	VIA	CS	100	361	16.62	8	Low
6	Gebrehiwot H2017 [14]	Ethiopia	VIA	CS	100	435	20.23	9	Very low
7	Gedefaw A, 2013 [42]	Ethiopia	VIA	CS	100	448	22.10	9	Very low
8	Gessesse Z, 2015 [15]	Ethiopia	VIA	CC	100	348	NA	8	Low
9	Getinet M, 2015 [37]	Ethiopia	Pap smear/cytology	CS	98.5	197	17.77	8	Low
10	Guthrie BL, 2020 [44]	Kenya	HPV DNA	CS	94.7	268	25.00	6	Moderate
11	Huchko MJ, 2014 [49]	Kenya	VIA	CS	100	3241	8.86	9	Very low
12	Izudi J, 2016 [17]	Uganda	VIA	RCT	100	995	6.33	9	Very low
13	Jolly PE, 2017 [18]	Eswatini	VIA	CS	100	109	22.94	7	Low
14	Kafuruki L, 2013 [55]	Tanzania	VIA	CS	100	355	26.76	8	Low
15	Kangethe JM, 2023 [19]	Kenya	VIA/VILI	CS	NA	224	21.43	6	Moderate
16	Kapiga SH, 1999 [56]	Tanzania	Pap smear/cytology	NA	99.28	686	2.92	7	Low
17	Kassa S, 2019 [38]	Ethiopia	VIA	CS	100	435	20.23	9	Very low
18	Kelly HA, 2017 [57]	South Africa	HPV DNA	PC	84.05	623	50.24	8	Moderate
19	Kirabira J, 2024 [52]	Uganda	Pap smear/cytology	CS	100	203	23.15	7	Low
20	Kiros M, 2021 [39]	Ethiopia	VIA	CS	NA	129	9.30	7	Low
21	Korn AK, 2022 [59]	Namibia	VIA	NA	NA	4805	16.69	6	Moderate
22	Lemma TM, 2024 [21]	Ethiopia	VIA	CS	100	257	15.95	7	Low
23	Lemu LG, 2021 [22]	Ethiopia	VIA	CS	97.80	444	18.69	8	Low
24	Liu E, 2016 [23]	Tanzania	Pap smear/cytology	CS	100	1365	8.72	10	Very low
25	Leroy V, 1999 [62]	Rwanda	Pap smear/cytology	PC	NA	103	24.27	5	Moderate
26	Mandiriri AM, 2020 [26]	Zimbabwe	VIA	PC	100	321	1.87	6	Moderate
27	McKenzie KP, 2011 [50]	Kenya	Pap smear/cytology	NA	100	267	46.07	5	Moderate
28	Memiah P, 2012 [51]	Kenya	VILI	NA	100	715	26.71	8	Low
29	Moodley JR, 2009 [58]	South Africa	Pap smear/cytology	CS	99	98	66.33	6	Moderate
30	Mugabe R, 2013 [54]	Uganda	VIA	CS	100	301	13.62	8	Low
31	Muia MO, 2021 [45]	Kenya	Pap smear/cytology	CS	96.25	385	7.79	8	Low
32	Mulugeta Y, 2022 [43]	Ethiopia	VIA	CS	91.26	334	16.47	9	Very low
33	Mungo C, 2024 [46]	Kenya	VIA	CS	NA	16,869	15.58	8	Low
34	MUTUKU OM, 2022 [47]	Kenya	Pap smear/cytology	PC	NA	400	7.75	6	Moderate
35	Naicker N, 2025 [24]	South Africa	HPV DNA	CS	99.23	258	33.33	8	Low
36	Njagi SK, 2021 [1]	Kenya	Pap smear/cytology	CS	NA	373	42.09	9	Very low
37	Odhiambo MA, 2016 [48]	Kenya	Pap smear/cytology	CS	100	210	10.00	8	Low
38	Oringo J, 2020 [53]	Uganda	Pap smear/cytology	CS	100	210	5.71	8	Low
39	Parham GP, 2006 [27]	Zambia	Pap smear/cytology	CS	100	150	76.00	7	Low
40	Phologolo T, 2023 [63]	Botswana	VIA	CS	NA	7,444	15.25	9	Very low
41	Sebastien TNJTIJAR, 2023 [60]	Namibia	Pap smear/cytology	CS	100	49	16.33	8	Low
42	Worku E, 2024 [25]	Ethiopia	VIA	CS	100	915	24.48	8	Low
43	Yonas M, 2025 [40]	Ethiopia	VIA	CS	98.70	397	20.40	9	Very low
44	Zelalem W, 2022 [41]	Ethiopia	VIA	CS	100	267	7.49	8	Low

HPV DNA: Human Papillomavirus Deoxyribonucleic Acid; Pap: Papanicolaou; VIA: Visual Inspection with Acetic Acid; VILI: Visual Inspection with Lugol's Iodine; NOS: Newcastle Ottawa Scale; NA: Not Applicable; CS: Cross-Sectional; CC: Case Control; RC: Randomized Control Trial; PC: Prospective Cohort.

### Prevalence of precancerous cervical lesions

This review revealed that the prevalence of PCCL among WLHIV in Eastern and Southern Africa varies from 1.87% (95% CI: 0.39, 3.51) to 76.00% (95% CI: 69.17, 82.83). The pooled prevalence of PCCL was 22.25% (95% CI: 19.57, 24.93), I^2^ = 98.43%, p < 0.001 (Fig 2). Of the pooled prevalence of PCCL, low-grade squamous intraepithelial lesions (CIN 1) accounted for the majority of PCCL (66%), while high-grade squamous intraepithelial lesions (CIN 2 and CIN 3) accounted for the remaining 34%.

**Fig 2:**
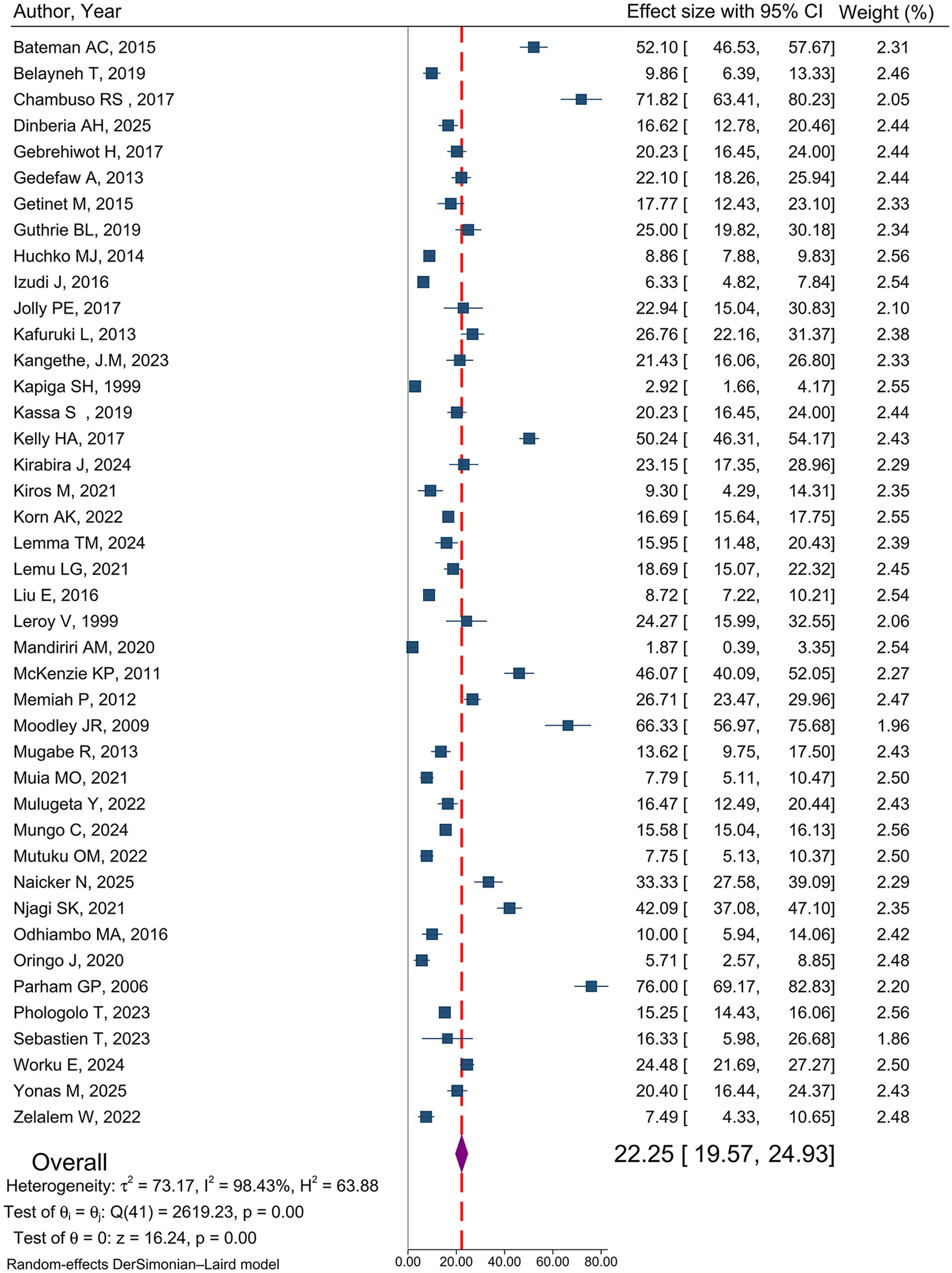
The prevalence of PCCL for each study and the pooled prevalence among WLHIV in Southern and Eastern Africa, 2025.

### Handling heterogeneity

Due to the heterogeneity observed, a random-effects meta-analysis model was employed to account for variability among the studies and provide a more reliable estimate. To explore potential causes of this heterogeneity, a subgroup analysis was carried out based on the geographical region of the studies, the year of publication, and the methods used for screening. Additionally, a sensitivity analysis was conducted.

The subgroup analysis of PCCL by geographical region was high among the studies conducted in Southern Africa, which was 31.08%. The lowest was observed in Eastern Africa, which was 20.66% (Fig 3). The reason for the difference between Southern and Eastern Africa is that Southern Africa has the highest HIV rates in the world. The difference in HIV infection rates increases PCCL by causing widespread immune system damage, which allows HPV to persist and facilitates the direct molecular actions of HIV proteins that accelerate cellular transformation.

**Fig 3:**
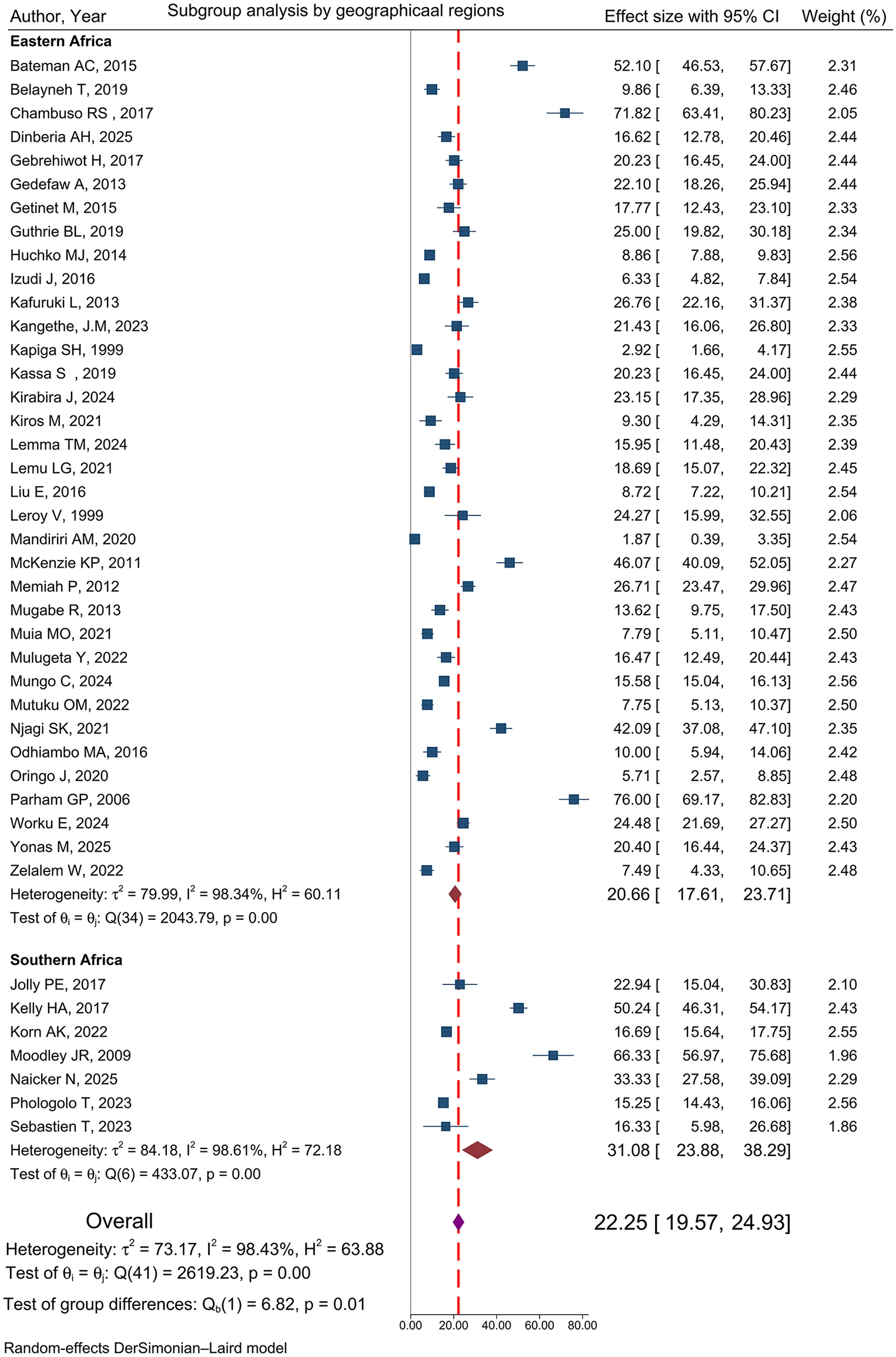
Subgroup analysis by the geographical region for PCCL and its associated factors among WLHIV, 2025.

A subgroup analysis by the study year of publication showed that studies published before 2019 reported a higher overall rate of PCCL, which was 28.08%. In contrast, studies published from 2020 to 2022 revealed a lower overall PCCL, which was 13.22% (Fig 4). The number of women screened before 2019, from 2020 to 2022, and post 2022 were 12052, 7668, and 27289, respectively. The lowest overall prevalence of PCCL and the lowest number of women screened occurred from 2020 to 2022. The reason behind it is due to the COVID-19 pandemic. The COVID-19 pandemic led to a significant decline in cervical cancer screenings, as clinics closed, healthcare resources diverted, and patients avoided care. It led to non-adherence to screening, which disproportionately affected under-represented populations (WLHIV) in lower socioeconomic regions (SEA). As a result, the occurrence of PCCL became lower. From 2023 to 2025, the prevalence of PCCL and the number of women screened increase to 19.67% and 27,289, respectively. The prevalence is adjusted once the pandemic has passed.

**Fig 4:**
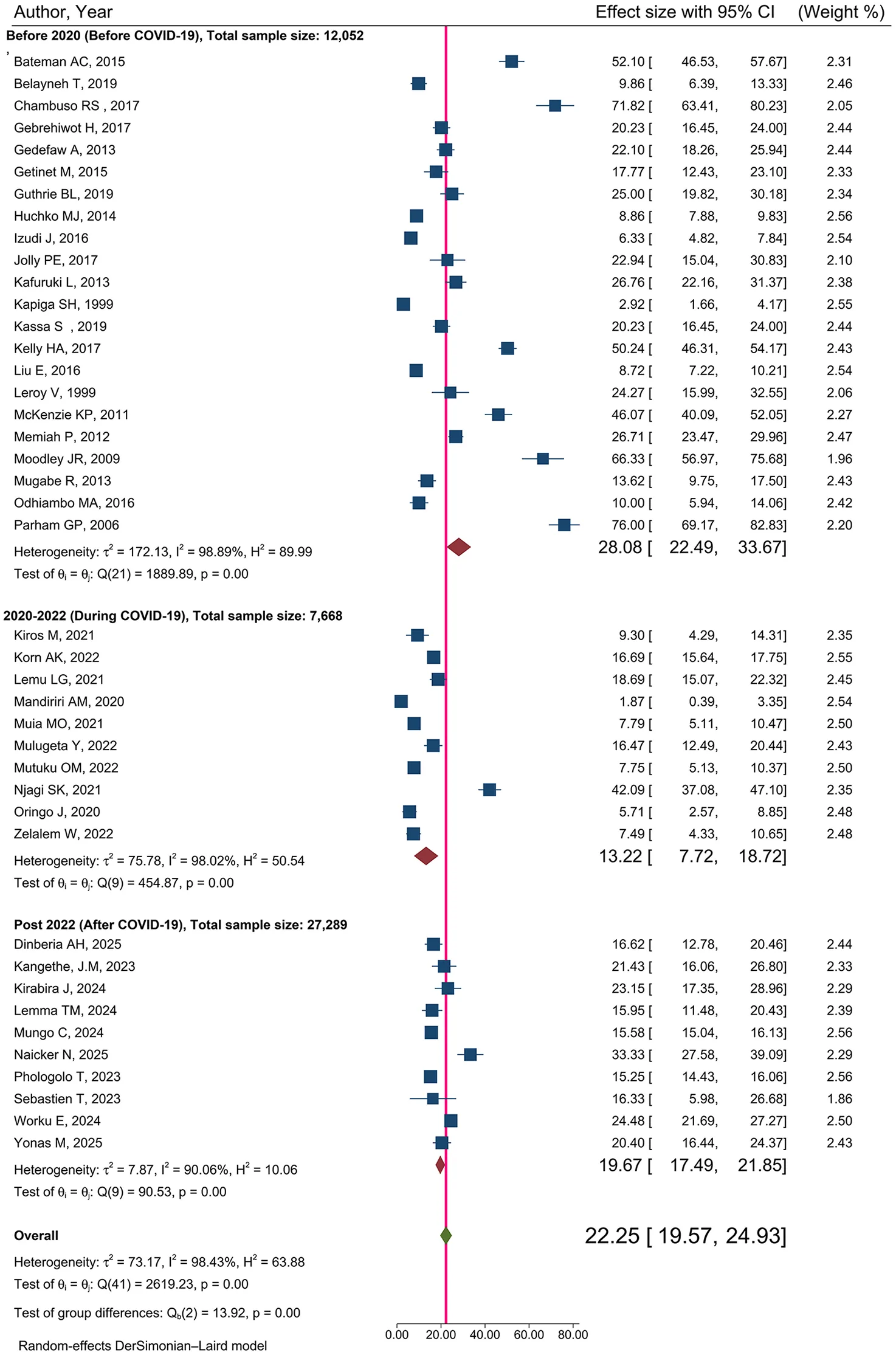
Subgroup analysis by the study year of publication for PCCL and associated factors among WLHIV, 2025.

Subgroup analysis by screening modalities revealed differences in the pooled prevalence of PCCL. In the random effects pooled subgroup meta-analysis, the overall prevalence of PCCL was 36.27%, 23.81%, and 19.65% for studies screened with HPV DNA, Pap smear/cytology, and VIA/VILA, respectively (Fig 5). **Here studies screened with** HPV DNA **showed the highest prevalence of** PCCL**, while studies screened with** VIA/VILA **showed the lowest. Studies screened with Pap smear/cytology are consistent with the overall prevalence of** PCCL **in this study. The possible explanations could be due to the sensitivity and specificity of the screening methods.** HPV DNA **tests are highly sensitive but lower in specificity when compared with the other methods. The** VIA/VILA **tests are highly operator-dependent and have poor sensitivity (< 70%) when compared to** HPV DNA**. Pap smear/cytology has moderate sensitivity (60–80%). The prevalence observed in those screened with cytology is moderate, accurately representing the overall pooled prevalence, as most global historical data relied on cytology. Furthermore, the high heterogeneity within each subgroup suggests that these prevalence estimates are influenced by variations in follow-up duration and geographical differences in** HPV **genotypes, rather than the screening methods alone.**

**Fig 5:**
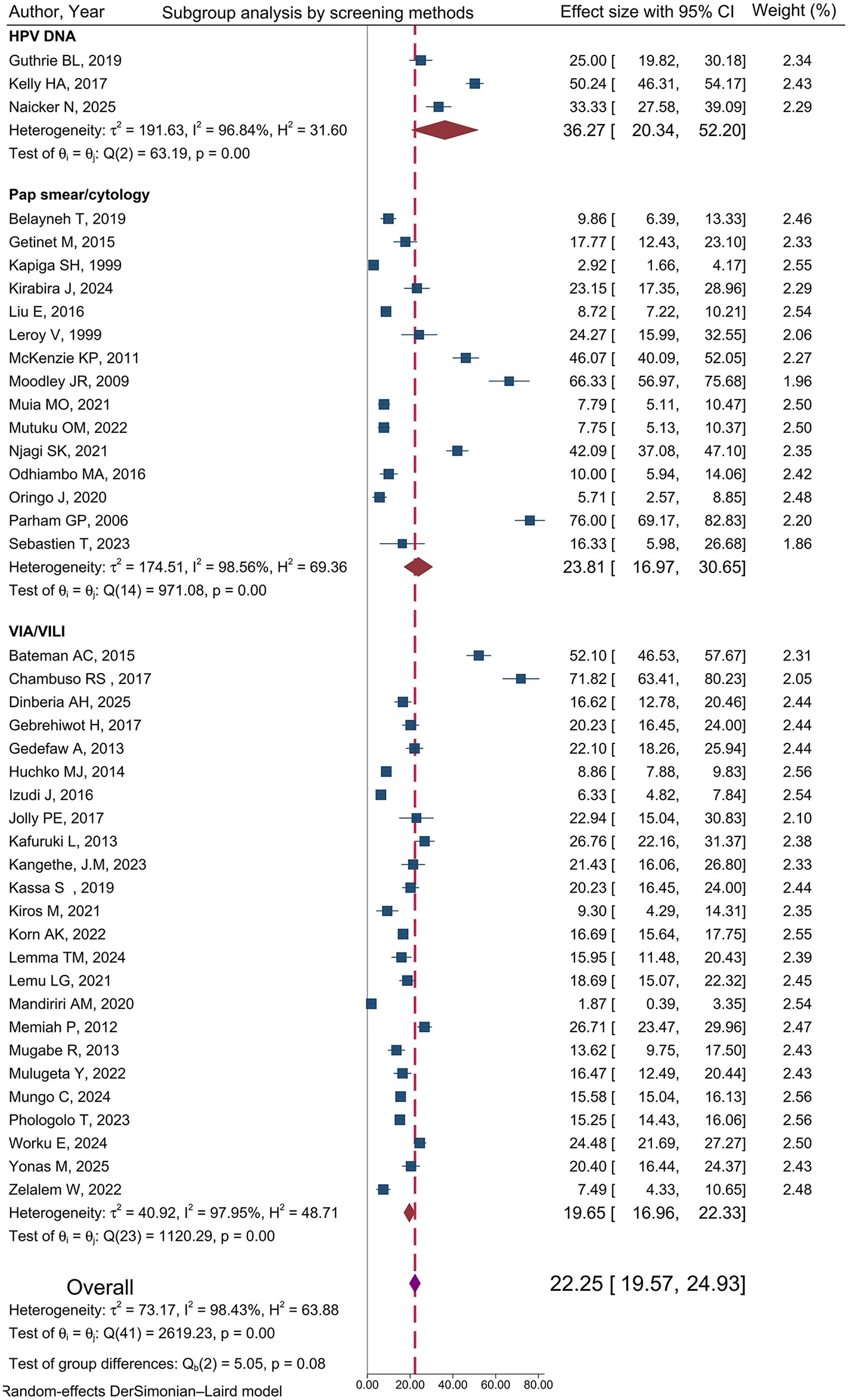
Subgroup analysis by screening modalities for precancerous cervical lesions and associated factors among WLHIV, 2025.

### Sensitivity analysis

To examine the robustness of the findings across the reviewed articles, a leave-one-out sensitivity analysis was conducted by removing one study at a time. This suggests that all the results were within the 95% CI, which means that none of the individual studies had a significant impact on the pooled prevalence of PCCL. This confirmed the validity of the pooled estimate (Fig 6).

**Fig 6:**
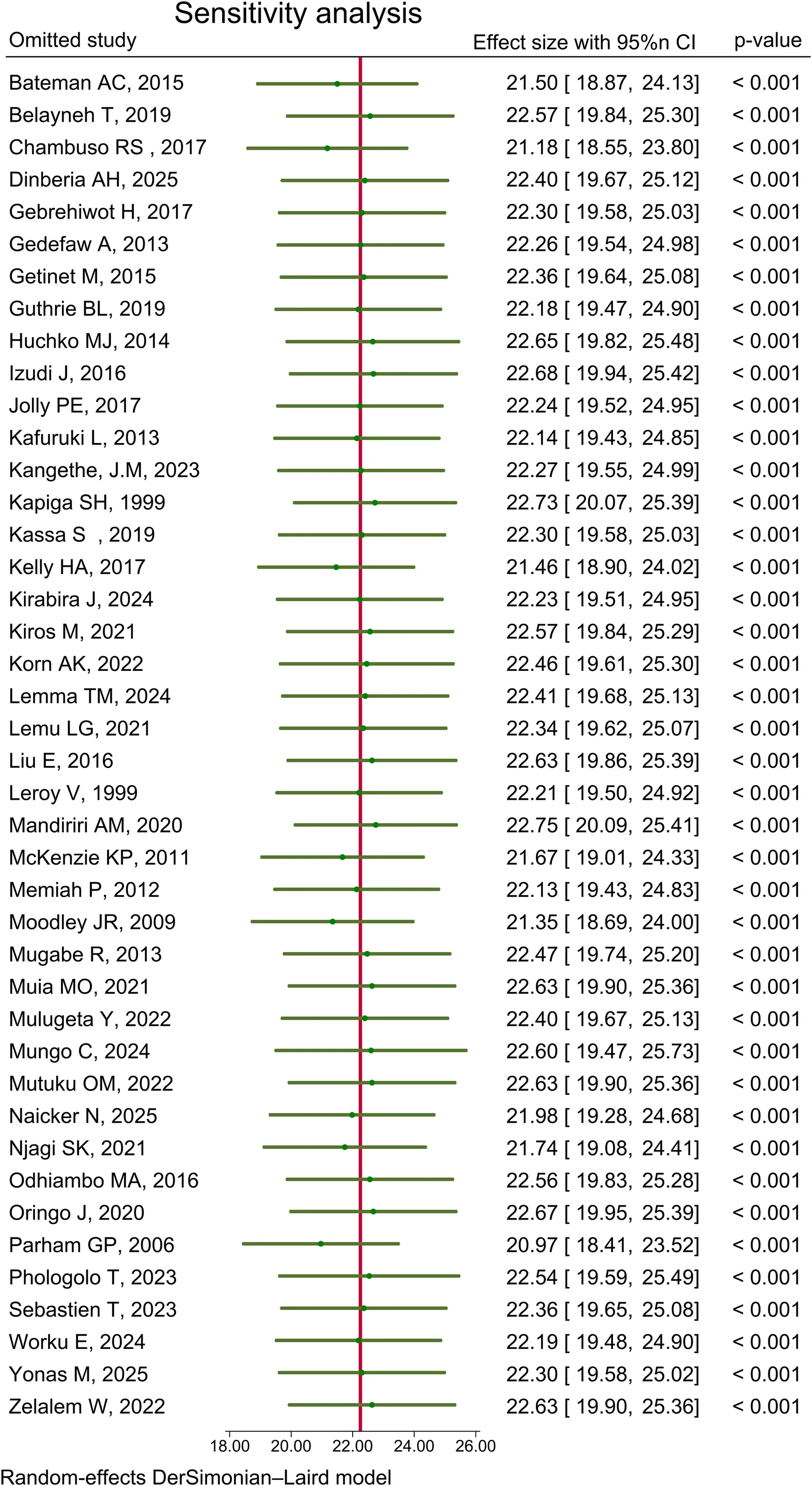
A sensitivity analysis to assess the robustness of studies for precancerous cervical lesions and associated factors among WLHIV, 2025.

There remained a high heterogeneity in PCCL even after subgroup and sensitivity analysis, suggesting that unmeasured factors, such as socio-economic characteristics, contextual differences, healthcare policies, and cultural norms, might have contributed and need to be explored further. Taken together, these factors may account for the significant heterogeneity in the prevalence of PCCL and the patterns in Eastern and Southern African countries and underline the need for tailored, context-specific interventions.

### Publication bias, trim and fill analysis

To assess publication bias, both the funnel plot and the Egger’s test were conducted in the meta-analysis. The funnel plot in this analysis demonstrated an uneven distribution, indicating the potential presence of publication bias among the studies used to estimate the pooled prevalence of PCCL, evidenced by the plot's asymmetry resulting from the unequal distribution of studies on its left and right sides (Fig 7a). Additionally, the regression-based Egger test for small-study effects (p < 0.001) produced statistically significant results, indicating the presence of publication bias. Therefore, a trim-and-fill analysis (Fig 7b) was performed to address the publication bias (Fig 7).

**Fig 7:**
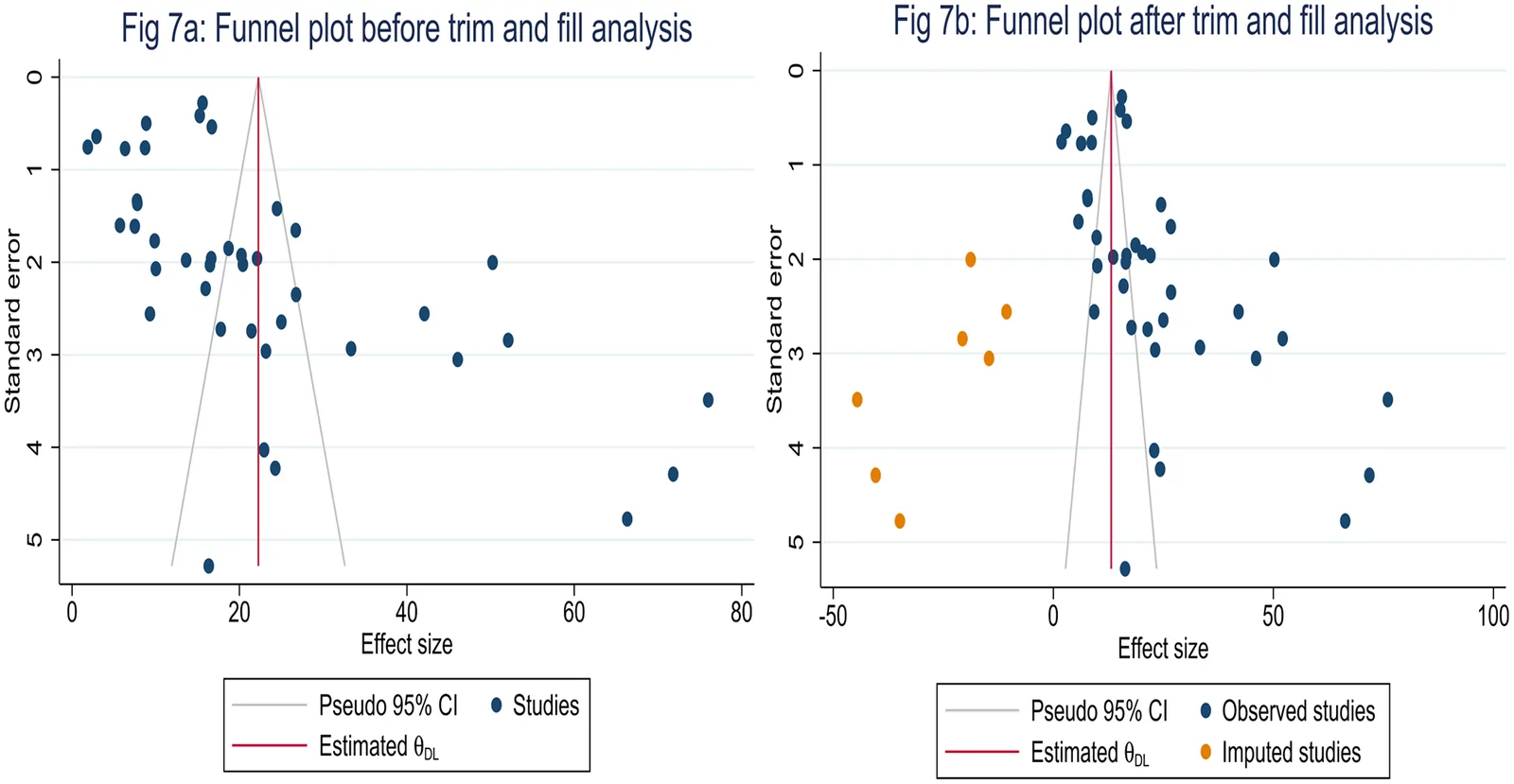
Funnel plot showing the publication bias on precancerous cervical lesions among WLHIV before adjustment “a” and after adjustment with trim and fill analysis “b” in Eastern and Southern Africa, 2025.

### Factors associated with precancerous cervical lesions among WLHIV

According to the review, significant associations (p < 0.05) between PCCL and various determinants were identified. These factors have been summarized with texts and forest plots. The lines and midpoints in the forest plots show each study’s OR with its 95% CI, and the diamond shape represents the pooled OR.

The pooled effects of seven studies [13,14,18,22,37,38,43] showed that those who commenced sexual intercourse before the age of 18 years were three (OR = 2.99, 95% CI: 2.34, 3.82, I^2^ = 23.7%) times more likely to develop PCCL in comparison to those who commenced sexual activity after the age of 18 years (Fig 8a). Likewise, three studies [11,37,54] indicated that the development of PCCL was nearly five times more likely among women whose parity was two or more when compared with those whose parity was less than two (OR = 4.97, 95% CI: 3.21, 7.69, I^2^ = 46.2%) (Fig 8b). Moreover, the meta-analysis results of the review from two studies [24,33] showed that the occurrence of PCCL among WLHIV who ever had a history of smoking was seven times higher than those who had not a history of smoking (OR = 7.01, 95% CI: 3.39, 14.49, I^2^ = 0) (Fig 8c). Furthermore, in Fig 8d, three studies [21,37,49] revealed that the odds of having PCCL were almost three (OR = 2.69, 95% CI: 1.85, 3.90, I^2^ = 39.0%) times higher among women who had ever taken oral contraceptive pills as compared to those who had not taken oral contraceptive pills (Fig 8).

**Fig 8:**
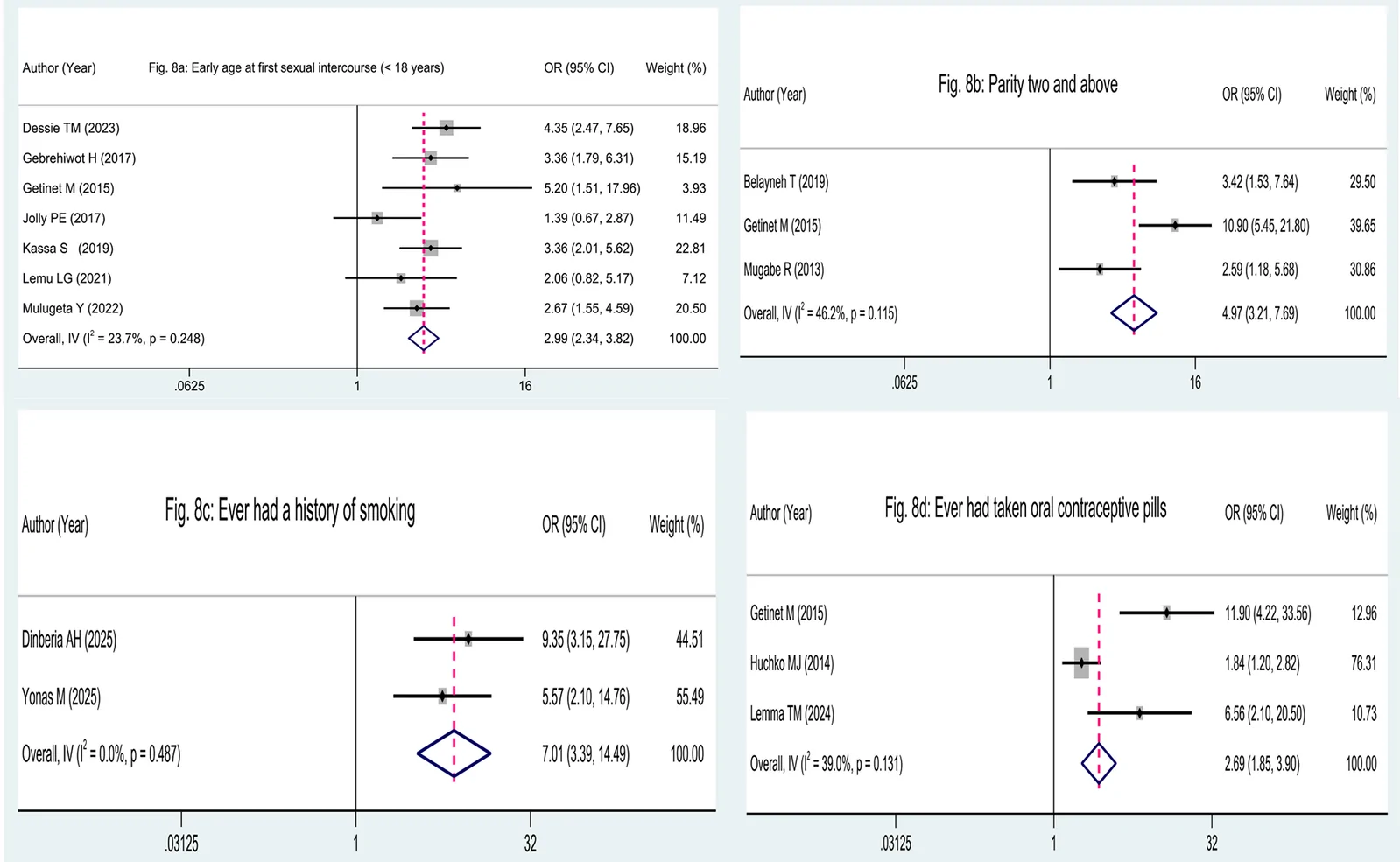
Forest plots of the odds ratios with the corresponding 95% CIs on the association between age, parity, smoking, oral contraceptive use, and PCCL among WLHIV in Southern and Eastern Africa, 2025.

The meta-analysis results of the review from fifteen studies [11,13,14,17,18,21,22,36–38,40–43,55] in Fig 9a showed that the odds of PCCL among women who had a history of STI were four times higher when compared with those who didn’t have a history of STI (OR = 3.93, 95% CI: 3.23, 4.78, I^2^ = 47%). Moreover, eleven studies [11,14,15,18,21,22,37,38,42,43,55] indicated that WLHIV who had two or more sexual partners in their lifetime (had ≥ 2 lifetime sexual partners) were almost three times (OR = 3.06, 95% CI: 2.45, 3.83, I^2^ = 0) more likely to have PCCL than women who had less than two sexual partners (Fig 9b). Furthermore, the meta-analysis results of the review from eleven studies [11,13,14,21,23,24,27,38,55,56,61] revealed that WLHIV who had a baseline CD4 count of < 200 cells/mm^3^ were 1.8 times more likely to develop PCCL than those who had a baseline CD4 count ≥ 200 cells/mm^3^ (OR = 1.79, 95% CI: 1.56, 2.05, I^2^ = 54.3%) (Fig 9c). Finally, in Fig 9d, five studies [22,25,42,49,51] indicated that WLHIV who had not initiated ART were two times more likely to be prone to PCCL when compared with those who had ever initiated ART (OR = 2.02, 95% CI: 1.56, 2.62, I^2^ = 0) (Fig 9).

**Fig 9:**
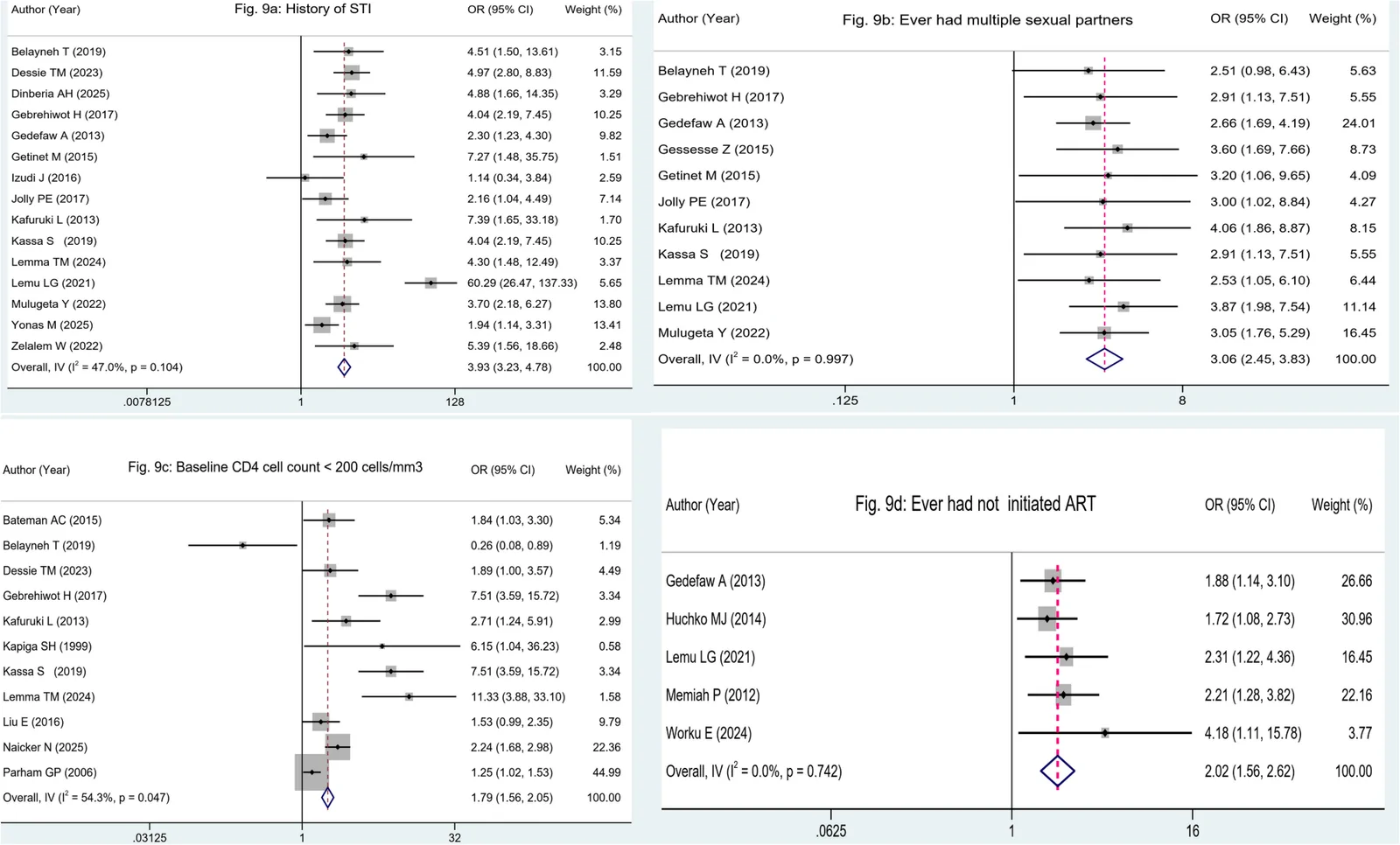
Forest plots of the odds ratios with the corresponding 95% CIs on the association between STI, multiple sexual partners, CD4 count, ART, and PCCL among WLHIV in Southern and Eastern Africa, 2025.

## Discussion

This SRMA aimed to synthesize the pooled prevalence of PCCL and associated factors among WLHIV in Eastern and Southern Africa. This review found a pooled prevalence of 22.25% (95% CI: 19.57, 24.93) PCCL among WLHIV, indicating about one-fourth of this high-risk population could develop cervical cancer if not treated early. The pooled prevalence of PCCL in the current study was higher than the prevalence reported in studies done in Ethiopia (13.4–15.16% [20,30,64], Latin America (4.1%) [56], Nigeria (9.1–16.67%) [65–67], and Togo (11.4%) [68]. The observed disparities between the current study and the previous studies may be due to the participant socio-economic and clinical characteristics and the study population [29,69]. The difference may be explained by the fact that some of the earlier studies referenced in this discussion included both HIV-positive and HIV-negative women [20,30,64], whereas the current study focused solely on HIV-positive women. WLHIV are more vulnerable to PCCL when compared with HIV-negative women. Factors such as compromised immunity and a higher risk of persistent HPV infection make WLHIV more susceptible to PCCL [2,7,9]. The findings of this review underscore the substantial gap to achieve the global and national commitment to eliminate cervical cancer [2]. The high prevalence of PCCL emphasizes the necessity of increasing cervical cancer screening, especially for underprivileged and marginalized groups, in addition to focused education, HPV vaccination, and cervical cancer prevention. Governments and other stakeholders should strengthen investments in HPV vaccination, screening, and timely treatment to reduce the cervical cancer burden.

The overall prevalence of PCCL in the current study was lower than the prevalence reported in Gambia (28.70%) [70]. The difference in the socioeconomic characteristics among the study participants could explain the disagreement [29,69]. The other possible explanation for the variation might be due to the difference in study design. The current study employs a meta-analysis, while the study conducted in Gambia utilized a cross-sectional study design [70]. The estimate in meta-analysis is an overall pooled, weighted measure derived from multiple studies, designed to provide the best summary effect, but the estimate in a cross-sectional study is a snapshot without synthesis from other data sources, which could overestimate the prevalence [71,72].

Regarding the determinants associated with the occurrence of PCCL, early age at first sexual intercourse (< 18 years) was significantly associated with PCCL. This finding is consistent with other studies conducted elsewhere [13,14,22,38,43,73]. The reason could be due to the fact that at this age cervical tissue undergoes physiological alterations, the transformation zone on the ectocervix is enlarged, and exposure to HPV during this period may promote infection, potentially making this area more susceptible to the development of dysplasia, a precursor to cervical squamous carcinoma. Moreover, an earlier initiation of sexual activity implies a prolonged duration of sexual exposure, which increases the likelihood of developing PCCL and cervical cancer [74,75].

The likelihood of PCCL was higher among WLHIV who had multiple sexual partners. The finding is comparable with a review conducted in Ethiopia [29]. The likelihood of PCCL was higher among WLHIV who had multiple sexual partners. The finding is comparable with a review conducted in Ethiopia [29]. The possible explanation is that women with multiple sexual partners are at a higher risk of PCCL, as having more sexual partners’ increase the likelihood of acquiring HPV infection. Additionally, the odds of PCCL were greater among WLHIV who had STIs compared to those without STIs. The finding is consistent with other reviews [29]. Evidence indicated that cervical cancer is caused by a viral infection, which is transmitted through sexual contact [5]. Often, patients with STIs have a history of multiple sexual partners, which increases their exposure to HPV. Sexually transmitted infections like HPV are the primary cause of PCCL, with sexual transmission being the main route of infection that initiates the carcinogenic process in the cervix [76,77].. In order to reduce the occurrence of PCCL, health professionals who regularly treat HIV-positive women should recommend cervical cancer screening and STI tests in addition to counselling them not to have multiple sexual partners.

WLHIV who had a baseline CD4 count of < 200 cells/mm^3^ were more likely to develop PCCL than those who had a baseline CD4 count ≥ 200 cells/mm^3^. This finding was comparable with other studies conducted elsewhere [5,78]. The possible explanation could be due to low CD4 counts, which indicate weakened immunity. Immunosuppression reduces the body’s ability to clear high-risk HPV infections. ART helps to raise CD4 counts, restore immune function, and reduce the risk and progression of PCCL [79,80] and should be given to all WLHIV.

The occurrence of PCCL among WLHIV who ever had a history of smoking was higher than those who did not smoke. This finding is similar to other studies that show smoking is strongly associated with an increased risk of developing PCCL [36,40]. The possible explanation could be smoking weakens the immune system’s ability to clear HPV infection and exposes cervical cells to carcinogens, causing DNA damage. It also contributes to the persistence and progression of HPV infection [81,82].

The odds of having PCCL were higher among WLHIV who had ever taken oral contraceptive pills. Earlier studies have shown similar findings [21,37]. The increased risk may be due to hormonal effects that enhance the persistence of high-risk HPV infection and cause changes in the cervical cells induced by oral contraceptive pills. Regular cervical cancer screening remains important for oral contraceptive pill users to detect and manage PCCL early, and alternative family planning should be given for those women [16,83].

WLHIV who had not taken ART were prone to PCCL when compared with those who had ever taken ART. This is consistent with the previous studies [22,25]. The possible elucidation could be due to the fact that being on ART enhances women’s immune response and reduces the likelihood of developing PCCL [84]. It can also be explained that ART lowers HPV acquisition and increases clearance via immune reconstitution [85]. This finding supports the current practice of early ART commencement for all HIV-positive individuals, irrespective of their CD4 levels and WHO clinical stage [86]. Early HIV testing, ART initiation, and scale-up of high-performance screening tests for WLHIV should be prioritized. Although the aforementioned factors were a crucial element of the PCCL, its preventive measures were overlooked by WLHIV and the country’s government of SEA.

### Strengths and limitations of the study

This study is the first to synthesize multiple primary studies from Southern and Eastern Africa to provide comprehensive estimates of PCCL and its associated factors among WLHIV. Additionally, the large sample size that spans the entire high-risk region enhances the generalizability and robustness of the findings. Despite these strengths, a dearth of studies from some countries restricts country-specific estimates and may not capture diverse local contexts. Moreover, there was a noticeable heterogeneity between the studies. To account for the variability among the studies, a random-effects model was employed, which provides more balanced and generalized estimates across variable studies and assumes multiple true effect sizes. A subgroup analysis and sensitivity were also performed in order to identify the possible reasons for the heterogeneity.

### Implications for policy, practice and future research

Findings from this study may have implications for health policy decisions, guideline development, and resource allocation to ultimately reduce the incidence of invasive cervical cancer and will provide better guidance for future interventions. This study underscores the burden of HPV disease in WLHIV who did not benefit from HPV vaccination. Access to HPV vaccines with expanded HPV type coverage is needed to ensure vaccination has a substantial impact on cervical cancer incidence and disease burden in the region. Continued and growing funding will be critical for building the resilient services that are needed for early diagnosis and treatment.

## Conclusion

The pooled prevalence of PCCL among WLWHV in Southern and Eastern Africa was high. Age, parity, sexual partner, STIs, smoking, oral contraceptives, CD4 count, and ART were factors positively associated with PCCL. Therefore, stakeholders should implement interventions to avert future incidents of PCCL and address the identified factors. Context-specific interventions like expanding awareness, building quality, culturally appropriate, decentralized screening infrastructures with health workforce capability, and focusing on socio-cultural barriers across regions are necessary to reduce PCCL.

## Supporting information

S1 Table A 27-item Preferred Reporting Items for Systematic Reviews and Meta-Analyses (PRISMA 2020) checklist. (DOCX)

S2 Table Search strings used for comprehensive search and the corresponding numbers in the databases. (DOCX)

S3 Table Newcastle – Ottawa Quality Assessment Scale. (DOCX)

S4 Table List of excluded studies based on the full text. (DOCX)

## Abbreviations

OR: Odds Ratio
ART: Antiretroviral Therapy
CD4: Cluster of Differentiation 4
CI: Confidence Interval
HPV: Human Papillomavirus
HIV: Human Immunodeficiency Virus
LMICs: Low-and Middle-Income Countries
NOS: Newcastle‒Ottawa Scale
PCCL: Precancerous Cervical Lesions
STI: Sexual Transmitted Infections
SEA: Southern and Eastern Africa
SSA: sub-Saharan Africa
SRMA: Systematic Review and Meta-Analysis
WHO: World Health Organization
WLHIV: Women Living with HIV
