# Supplementary material for: Precancerous cervical lesions and associated factors among HIV-positive women in Southern and Eastern African countries: A systematic review and meta-analysis
Source: PLoS One. 2026 Oct 23;21(10):e0361185. doi: 10.1371/journal.pone.0361185 (PMC13693988; doi:10.1371/journal.pone.0361185)
Supplement: Supplementary file 2 [file pone.0361185.s002.docx]

**S2 Table: Search strings used for a comprehensive search and the corresponding numbers for the databases**

Data bases, Search results and search strings

**PubMed = 638**

((("precancerous" (MeSH), OR (precancerous cervical lesion) OR (cervical lesion)), OR ("Cervical Intraepithelial Neoplasia" (MeSH)) OR "Cervical caner" AND (Determinants (MeSH)) OR (Predictors) OR ("Associated Factors") OR ("Risk Factors"[MeSH Terms]) OR (Barriers) AND (Burundi) OR (Botswana) OR (Comoros) OR (Djibouti) OR (Eritrea) OR (Eswatini) OR (Ethiopia) OR (Kenya) OR (Lesotho) OR (Madagascar) OR (Malawi) OR (Mauritius) OR (Mozambique) OR (Namibia) OR (Rwanda) OR (Seychelles)) OR (Somalia) OR ("South Africa") ("South Sudan") OR (Swaziland) OR (Tanzania) OR (Uganda) OR (Zambia) OR (Zimbabwe)) OR ("Eastern Africa") OR ("Southern Africa") AND ("HIV positive") "HIV/AIDS" OR ("HIV Infections" (MeSH Terms)) OR ("HIV Sero positivity") OR ("Women living with HIV" (MeSH Terms))

**NB:For the above search strategy filters applied to full text.**

**EMBASE: 316**

(precancerous cervical lesion AND (Burundi) OR (Botswana) OR (Comoros) OR (Djibouti) OR (Eritrea) OR (Eswatini) OR (Ethiopia) OR (Kenya) OR (Lesotho) OR (Madagascar) OR (Malawi) OR (Mauritius) OR (Mozambique) OR (Namibia) OR (Rwanda) OR (Seychelles)) OR (Somalia) OR ("South Africa") ("South Sudan") OR (Swaziland) OR (Tanzania) OR (Uganda) OR (Zambia) OR (Zimbabwe)) AND ('women living with hiv') AND [female]/lim AND [humans]/lim AND [english]/lim AND [embase]/lim AND 'human immunodeficiency virus infection'/dm AND 'human'/de AND 'article'/it

**SCOPUS: 247**

" Prevalence of precancerous cervical lesion" AND "Women living with HIV" OR "HIV positive women" AND determinants OR predictors OR "Risk factor" OR "Associated factors" AND Burundi OR Botswana OR Comoros OR Djibouti OR Eritrea OR Eswatini OR Ethiopia OR Kenya OR Lesotho OR Madagascar OR Malawi OR Mauritius OR Mozambique OR Namibia OR Rwanda OR Seychelles OR Somalia OR "South Africa" OR "South Sudan" OR Swaziland OR Tanzania OR Uganda OR Zambia OR Zimbabwe AND ( LIMIT-TO ( DOCTYPE , "ar" ) ) AND ( LIMIT-TO ( EXACTKEYWORD , "Female" ) ) AND ( LIMIT-TO ( SRCTYPE , "j" ) )

**HINARI through research4life: 66**

((Precancerous cervical lesions) OR (Cervical intraepithelial neoplasia)) AND (Determents) OR (Barriers) OR (Associated factors) AND ((Women living with HIV) OR (HIV positive women)) AND (Eastern Africa)

**ScinceDirect : 119**

Precancerous cervical lesions and associated factors among HIV‐positive women in Eastern and Southern African countries

**Google Scholar: 505**

"Precancerous cervical lesions" AND Determinants OR “Associated factors” OR "Risk factors" AND Burundi OR Botswana OR Comoros OR Djibouti OR Eritrea OR Eswatini OR Ethiopia OR Kenya OR Lesotho OR Madagascar OR Malawi OR Mauritius OR Mozambique OR Namibia Rwanda OR Seychelles OR Somalia OR "South Africa" OR "South Sudan" OR Swaziland Tanzania OR Uganda OR Zambia OR Zimbabwe OR “Eastern Africa”OR “Southern Africa” AND "Women living with HIV"
