# Supplementary material for: Precancerous cervical lesions and associated factors among HIV-positive women in Southern and Eastern African countries: A systematic review and meta-analysis
Source: PLoS One. 2026 Oct 23;21(10):e0361185. doi: 10.1371/journal.pone.0361185 (PMC13693988; doi:10.1371/journal.pone.0361185)
Supplement: Supplementary file 3 [file pone.0361185.s003.docx]

| **S3 Table: Newcastle - Ottawa Quality Assessment Scale** | | | | | | | | | | |
| --- | --- | --- | --- | --- | --- | --- | --- | --- | --- | --- |
| **Authors’ names** | **Year of publication** | **Selection (maximum 5 scores)** | | | | **Comparability (Max. 2 scores)** | | **Outcome (Max. 3 scores)** | | **Total score** |
|  |  | Representativeness of the sample (1*) | Sample size (1*) | Non-response rate  (1*) | Ascertainment of exposure (2*) | The study controlled potential confounders (1*) | The study controlled any additional factor (1*) | Independent blind assessment (2*) | Statistical test (1*) | **10 points** |
| Bateman AC | 2015 | * |  | * | * | * | * | * | * | 7 |
| Belayneh T | 2019 | * |  |  | ** | * | * | ** |  | 8 |
| Chambuso RS | 2017 | * |  | * | * | * | * | * | * | 7 |
| Dessie TM | 2025 | * |  | * | ** | * | * | * | * | 9 |
| Dinberia AH | 2025 | * |  | * | ** | * | * | ** | * | 8 |
| Gebrehiwot H | 2017 | * | * | * | * | * | * | ** | * | 9 |
| Gedefaw A | 2013 | * | * | * | * | * | * | ** | * | 9 |
| Gessesse Z | 2015 | * |  | * | ** | * | * | * | * | 8 |
| Getinet M | 2015 | * |  | * | ** | * | * | * | * | 8 |
| Guthrie BL | 2020 |  |  | * | * | * | * | * | * | 6 |
| Huchko MJ | 2014 | * | * | * | ** | * | * | * | * |  |
| Izudi J | 2016 | * | * | * | ** | * | * | * | * | 9 |
| Jolly PE | 2017 |  |  | * | ** | * |  | ** | * | 7 |
| Kafuruki L | 2013 | * |  | * | * | * | * | ** | * | 8 |
| Kangethe JM | 2023 |  |  |  | ** | * | * | * | * | 6 |
| Kapiga SH | 1999 |  | * | * | * | * | * | * | * | 7 |
| Kassa S | 2019 | * | * | * | ** | * | * | * | * | 9 |
| Kelly HA, | 2017 | * |  | * | ** | * | * | * | * | 8 |
| Kirabira J | 2024 | * |  | * | * | * | * | ** |  | 7 |
| Kiros M | 2021 | * |  | * | ** | * |  | * | * | 7 |
| Korn AK | 2022 | * | * |  | ** |  |  | ** |  | 6 |
| Lemma TM | 2024 | * |  |  | ** | * | * | * | * | 7 |
| Lemu LG, | 2021 | * |  | * | ** | * | * | * | * | 8 |
| Liu E | 2016 | * | * | * | ** | * | * | ** | * | 10 |
| Leroy V | 1999 | * |  | * | * | * |  | * |  | 5 |
| Mandiriri AM | 2020 | * |  | * | ** |  |  | ** |  | 6 |
| McKenzie KP | 2011 | * |  | * | * |  |  | * | * | 5 |
| Memiah P | 2012 | * | * | * | * | * | * | * | * | 8 |
| Moodley JR | 2009 |  | * | * | * | * | * |  | * | 6 |
| Mugabe R | 2013 |  | * | * | ** | * | * | * | * | 8 |
| Muia MO | 2021 | * |  | * | ** | * | * | * | * | 8 |
| Mulugeta Y | 2022 |  | * | * | ** | * | * | ** | * |  |
| Mungo C | 2024 | * |  | * | ** | * | * | * | * | 8 |
| MUTUKU OM | 2022 | * |  |  | ** | * |  | ** |  | 6 |
| Naicker N | 2025 | * |  | * | ** | * | * | * | * | 8 |
| Njagi SK | 2021 | * | * | * | ** | * |  | ** | * | 9 |
| Odhiambo MA | 2016 | * |  | * | ** | * | * | ** |  | 8 |
| Oringo J | 2020 | * |  | * | ** | * | * | * | * | 8 |
| Parham GP | 2006 |  | * |  | ** | * | * | * | * | 7 |
| Phologolo T | 2023 | * | * |  | ** | * | * | ** | * | 9 |
| Sebastien TNJTIJAR | 2023 | * |  | * | ** | * | * | ** |  | 8 |
| Worku E | 2024 | * |  | * | ** | * | * | * | * | 8 |
| Yonas M | 2025 | * | * |  | ** | * | * | ** | * | 9 |
| Zelalem W | 2022 | * |  | * | ** | * | * | * | * | 8 |

**Note: Cross-sectional Studies: Very Good Studies: 9-10 points; Good Studies: 7-8 points; Satisfactory Studies: 5-6 points; Unsatisfactory Studies: 0 to 4 points. One star stands for 1 score (*****), two star (******) stands for 2 score**

**Criteria for Evaluations**

A. **NEWCASTLE - OTTAWA QUALITY ASSESSMENT SCALE (adapted for cross sectional studies)**

**Selection: (Maximum 5 scores)**

**1) Representativeness of the sample: (1 score)**

a) Truly representative of the average in the target population (consecutive or random sampling). 1 score
b) Somewhat representative of the average in the target population (non-random sampling) 1 score
c) Selected group of subjects. 0 score
d) No description of the sampling strategy. 0 score

**2) Sample size: (1 score)**

a) Justified and satisfactory (≥ 400). 1 score
b) Not justified (< 400). 0 score

**3) Non-Response rate: (1 score)**

a) The response rate is satisfactory ( ≥ 90%). 1 score
b) The response rate is unsatisfactory (< 90%) or no description. 0 score
c) No description of the response rat. 0 score

**4) Ascertainment of the exposure (risk factor):** **(2 score)**

a) Validated measurement tool. 2 scores
b) Non-validated measurement tool, but the tool is available or described. 1 score
c) No description of the measurement tool. 0 score

**Comparability: (Maximum 2 scores)**

1) The potential confounders were controlled by subgroup analysis or multivariable analysis.

a) The study controlled potential confounders. 1 score
b) The study controlled any additional factor. 1 score
c) The study does not investigate potential confounders. 0 score

**Outcome: (Maximum 3 scores)**

**1) Assessment of the outcome:**

a) Independent blind assessment. 2 scores
b) Record linkage. 1 scores
c) Self-report. 1 score
d) No description. 0 score

**2) Statistical test:**

a) The statistical test used to analyze the data is clearly described and appropriate. 1 score
b) The statistical test is not appropriate, not described, or incomplete. 0 score
B. **NEWCASTLE - OTTAWA QUALITY ASSESSMENT SCALE COHORT STUDIE**S

**Note: A study can be awarded a maximum of one star for each numbered item within the Selection and Outcome categories. A maximum of two stars can be given for Comparability**

**Selection** (**maximum** **4 stars**)

1) **Representativeness of the exposed cohort**

a) truly representative of the average adults with DM in the community **(one star)**
b) somewhat representative of the average adults with DM in the community **(one star)**
c) selected group of volunteer subjects
d) no description of the derivation of the cohort

2) **Selection of the non-exposed cohort**

a) drawn from the same community as the exposed cohort **(one star)**
b) drawn from a different source
c) no description of the derivation of the non-exposed cohort

3) Ascertainment of exposure

a) secure record (eg surgical records) **(one star)**
b) structured interview **(one star)**
c) written self-report
d) no description

4) Demonstration that outcome of interest was not present at start of study

a) yes **(one star)**
b) no

**Comparability** (**maximum** 2 **stars**)

1) Comparability of cohorts based on the design or analysis

a) Study controls for confounding (multivariable analysis). 1 star
b) Study controls for any additional factor. 1 star
c) Cohorts are not comparable based on the design or analysis controlled for confounders

**Outcome** (**maximum** **3 stars**)

1) Assessment of outcome

a) Independent blind assessment. 1 star
b) record linkage (**one star**)
c) self-report
d) no description

2) Was follow-up long enough for outcomes to occur

a) yes (select an adequate follow up period for outcome of interest) **(one star)**
a) no

3) Adequacy of follow up of cohorts

a) complete follow up - all subjects accounted for **(one star)**
b) subjects lost to follow up unlikely to introduce bias - small number lost <15% or description of those lost suggested no different from those followed **(one star)**
c) Follow up rate less than 80% and no description of those lost no statement
d) No statement
