# Supplementary material for: Precancerous cervical lesions and associated factors among HIV-positive women in Southern and Eastern African countries: A systematic review and meta-analysis
Source: PLoS One. 2026 Oct 23;21(10):e0361185. doi: 10.1371/journal.pone.0361185 (PMC13693988; doi:10.1371/journal.pone.0361185)
Supplement: Supplementary file 4 [file pone.0361185.s004.docx]

**S4 Table: List of excluded studies based on the full text**

| N^o^ | Author, year | Title | Reference URL or DOI | Main reason for exclusion |
| --- | --- | --- | --- | --- |
| 1. | Kufa, 2023 | Prevalence of cervical high-risk human papillomavirus among Zimbabwean women living with HIV | <https://doi.org/10.4102/sajhivmed> | No outcome of interest |
| 2. | Keto, 2022 | Prevalence of Premalignant Cervical Lesions and Associated Risk Factors among Women Aged 21 - 69 Years at Dodoma Regional Referral Hospital, Tanzania | <https://www.proquest.com/openview/c50e55bdcde1c400fc9ef722f3fc78de/1?pq-origsite=gscholar&cbl=2026366&diss=y> | The target population did not consist of only WLHIV |
| 3. | [Getinet](https://www.tandfonline.com/author/Getinet%2C+Mamaru), 2021 | Precancerous Lesions of the Cervix and Associated Factors among Women of East Gojjam, Northwest Ethiopia, 2020 | <https://www.tandfonline.com/doi/full/10.2147/CMAR.S338177> | The target population are not women living with HIV |
| 4. | Lema, 2023 | Epidemiology of Pre-cancerous Cervical Lesions Among Women in Adama, Ethiopia | <https://www.researchsquare.com/article/rs-5376561/v1> | The target population are not women living with HIV |
| 5. | Teklehaimanot, 2024 | Precancerous lesion determinants in women attending cervical cancer screening at public health facilities in North Shoa Zone, Amhara, Ethiopia: an unmatched case-control study | <https://link.springer.com/article/10.1186/s12905-024-03113-z> | The target population are not women living with HIV |
| 6. | Teame, 2018 | Factors associated with cervical precancerous lesions among women screened for cervical cancer in Addis Ababa, Ethiopia: A case control study | <https://journals.plos.org/plosone/article?id=10.1371/journal.pone.0191506> | The target population are not women living with HIV |
| 7. | Dibisa, 2022 | Precancerous Lesion of the Cervix and Associated Factors Among Women of West Wollega, West Ethiopia, 2022 | <https://journals.sagepub.com/doi/full/10.1177/10732748221117900> | The target population are not women living with HIV |
| 8. | Misgina, 2021 | Prevalence of precancerous cervical lesion and associated factors among women in North Ethiopia | <https://academicjournals.org/journal/JPHE/article-full-text/394E9DC62810> | The target population are not women living with HIV |
| 9. | Temesgen, 2019 | Proportions of Pre-Cancerous Cervical Lesions and Its Associated Factors among Women Clients in the Age Group of 30-49yrs in Gynecology Ward of Dessie Referral Hospital and FGAE, North-East Ethiopia, 2016 | <https://www.researchgate.net/publication/365490259> | The target population are not women living with HIV |
| 10. | Taye, 2021 | Risk factors of precancerous cervical lesions: The role of women’s socio-demographic, sexual behavior and body mass index in Amhara region referral hospitals; case-control study | <https://journals.plos.org/plosone/article?id=10.1371/journal.pone.0249218> | The target population are not women living with HIV |
| 11. | Ssedyabane, 2024 | Prevalence of cervical intraepithelial lesions and associated factors among women attending a cervical cancer clinic in Western Uganda; results based on Pap smear cytology | <https://doi.org/10.1038/s41598-022-18978-z> | The target population are not women living with HIV |
| 12. | Gemechu, 2024 | Prevalence Of High-Risk Human Papillomavirus, Precancerous Lesion And Associated Factors Among Women Attending Gynecologic Unit Of Ambo General Hospital, West Shewa, Oromia, Ethiopia | <https://irlib.ambou.edu.et/xmlui/handle/123456789/3969> | The target population are not women living with HIV |
| 13. | Wakwoya, 2020 | Prevalence of Abnormal Cervical Lesions and Associated Factors Among Women in Harar, Eastern Ethiopia | <https://www.tandfonline.com/doi/full/10.2147/CMAR.S279712> | The target population are not women living with HIV |
| 14. | Christensen, 2023 | Fighting cervical cancer in Africa: a cross-sectional study on prevalence and risk factors for precancerous lesions in rural Uganda | https://www.sciencedirect.com/science/article/pii/S0033350623003608 | The target population are not women living with HIV |
| 15. | Aredo, 2021 | Knowledge of cervical cancer screening and associated factors among women attending maternal health services at Aira Hospital, West Wollega, Ethiopia | <https://creativecommons.org/licenses/by-nc/4.0/> | The target population are not women living with HIV |
| 16. | KINOTI, 2023 | HUMAN PAPILLOMAVIRUS PREVALENCE, GENOTYPES, AND FACTORS ASSOCIATED WITH CERVICAL ABNORMALITIES AMONG HIVPOSITIVE AND NEGATIVE WOMEN IN EASTERN AND CENTRAL REGIONS, KENYA | <https://irlibrary.ku.ac.ke/server/api/core/bitstreams/d9dfd8c6-b191-420f-b566-f31fcba791de/content> | No outcome of interest |
| 17. | Minwuyelet, 2025 | Detection of pre-cervical cancer and its associated factors among cervical cancer suspected women in Bichena primary hospital in Amhara region, Ethiopia | <https://www.researchsquare.com/article/rs-6534651/v1> | The target population are not women living with HIV |
| 18. | Ogu, 2019 | Prevalence and Risk Factors of Cervical Dysplasia among Human  Immunodeficiency Virus Sero-Positive Females on Highly Active Antiretroviral Therapy in Enugu | https://pmc.ncbi.nlm.nih.gov/articles/PMC6982680/ | The study was not conducted exclusively in SEA |
| 19. | Twaambo, 2015 | Predictors of abnormal cervical lesions among women(15-49 years old) in Zambia: A cross-sectoral study | <https://dspace.unza.zm/items/04a6aba6-b7c6-4425-9cf6-ce99cc5cf10d> | The target population are not women living with HIV |
| 20. | **Garoma**, 2021 | Precancerous Cervical Cancer Lesion and Associated Factors among Rural Women of Age 30 – 49 Years, in Sude District, Oromia Region, Ethiopia | <https://mail.ejrh.org/index.php/ejrh/article/view/423> | The target population are not women living with HIV |
| 21. | EMMANUEL, 2023 | HUMAN PAPILLOMAVIRUS TESTING FOR CERVICAL CANCER AMONG WOMEN LIVING WITH HIV AT THE CAPE COAST TEACHING HOSPITAL | <https://ir.ucc.edu.gh/xmlui> | The primary outcome of interest was not reported |
| 22. | Makuza, 2015 | Prevalence and risk factors for cervical cancer and pre-cancerous lesions in Rwanda | <http://www.panafrican-med-journal.com/content/article/22/26/full/> | No outcome of interest |
| 23. | Mcharo , 2021 | HPV Type Distribution in HIV Positive and Negative Women With or Without Cervical Dysplasia or Cancer in East Africa | doi: 10.3389/fonc.2021.763717 | No outcome of interest |
| 24. | MUSEZA, 2022 | PREVALENCE AND DETERMINANTS OF PRECANCEROUS LESIONS IN HPV-DNA POSITIVE WOMEN IN THE POPULATION  SOLUTIONS FOR HEALTH CERVICAL CANCER SCREENING  PROGAM IN ZIMBABWE |  | The target population are not women living with HIV |
| 25. | Rantshabeng, 2024 | High-risk human papillomavirus diversity  among indigenous women of western  Botswana with normal cervical cytology  and dysplasia | <https://doi.org/10.1186/s12879-024-10058-z> | No outcome of interest |
| 26. | Taku, 2021 | Distribution of Human Papillomavirus (HPV) Genotypes in HIV-Negative and HIV-Positive Women with Cervical Intraepithelial Lesions in the Eastern Cape Province, South Africa | https://www.mdpi.com/1999-4915/13/2/280 | No outcome of interest |
| 27. | **File,** 2021 | Precancerous Cervical Cancer Lesion and Associated Factors among Rural Women of Age 30 – 49 Years, in Sude District, Oromia Region, Ethiopia | <https://mail.ejrh.org/index.php/ejrh/article/view/423> | The target population are not women living with HIV |
| 28. | Fentie, 2020 | Factors affecting cervical cancer screening uptake, visual inspection with acetic acid positivity and its predictors among women attending cervical cancer screening service in Addis Ababa, Ethiopia | <https://doi.org/10.1186/s12905-020-01008-3> | No outcome of interest |
| 29. | Eilu, 2020 | Prevalence of cervical intraepithelial neoplasia and its associated factors among women attending healthcare services in Eastern Uganda | <https://academicjournals.org/journal/JCREO/article-full-text/4F13EA163589> | The target population are not women living with HIV |
| 30. | Alamirew, 2022 | Predictors of Precancerous Cervical Lesions Among Women Screened for Cervical Cancer in Bahir Dar Town, Ethiopia: A Case–Control Study | <https://www.tandfonline.com/doi/full/10.2147/CMAR.S258167> | The target population are not women living with HIV |
| 31. | Amilor, 2019 | Human Papillomavirus (HPV) Infection And Its Association With Cervical Dysplasia Among HIV Positive Women Attending Art Clinic, Korle-Bu Teaching Hospital | <http://ugspace.ug.edu.gh:8080/handle/123456789/40431> | The study was not conducted exclusively in SEA |
| 32. | Deksissa, 2015 | Prevalence and factors associated with VIA positive result among clients screened at Family Guidance Association of Ethiopia, south west area office, Jimma model clinic, Jimma, Ethiopia 2013: a cross-sectional study | <https://link.springer.com/article/10.1186/s13104-015-1594-x> | The target population are not women living with HIV |
| 33. | Michael, 2013 | Risk Factors for Cervical Precancer Lesions among Women Attending Cervical Cancer Screening Clinics in Harare, 2013 | <https://d1wqtxts1xzle7.cloudfront.net/81098225> | The target population are not women living with HIV |
| 34. | Derbie, 2022 | Histopathological profile of cervical punch biopsies and risk factors associated with high-grade cervical precancerous lesions and cancer in northwest Ethiopia | <https://pubmed.ncbi.nlm.nih.gov/36094938/> | The target population are not women living with HIV |
| 35. | Heard, 2015 | High Prevalence of Anal Human Papillomavirus-Associated Cancer  Precursors in a Contemporary Cohort of Asymptomatic HIV-Infected Women | <https://pubmed.ncbi.nlm.nih.gov/25645213/> | The study was not conducted exclusively in SEA |
| 36. | Gupta, 2022 | High Prevalence of Cervical High-Grade Lesions and High-Risk Human Papillomavirus Infections in Women Living with HIV: A Case for Prioritizing Cervical Screening in This Vulnerable Group | <https://pubmed.ncbi.nlm.nih.gov/35760059/> | No outcome of interest |
| 37. | Swai, 2021 | The association between human papillomavirus and cervical high-grade cytology among HIV-positive and HIV-negative Tanzanian women: A cross-sectional study | <https://pubmed.ncbi.nlm.nih.gov/33512002/> | No outcome of interest |
| 38. | [McClymont](https://pubmed.ncbi.nlm.nih.gov/?term=McClymont+E&cauthor_id=36702811), 2023 | HSV-2 infection and HPV incidence, persistence, and precancerous lesions in a cohort of HPV-vaccinated women living with HIV | <https://pubmed.ncbi.nlm.nih.gov/36702811/> | The study was not conducted exclusively in SEA |
| 39. | Hayumbu, 2021 | Cervical cancer and precancerous cervical lesions detected using visual inspection with acetic acid at Livingstone Teaching Hospital | <https://pubmed.ncbi.nlm.nih.gov/35178146/> | The study was not conducted exclusively in SEA |
| 40. | Wodajo, 2025 | Optimising the effectiveness of the see and treat program for cervical intracellular neoplasia among HIV-positive women, Addis Ababa Ethiopia | <https://pubmed.ncbi.nlm.nih.gov/40304663/> | No outcome of interest |
| 41. | Namale, 2021 | Visual inspection with acetic acid (VIA) positivity among female sex workers: a cross-sectional study highlighting one-year experiences in early detection of pre-cancerous and cancerous cervical lesions in Kampala, Uganda | <https://pubmed.ncbi.nlm.nih.gov/33975633/> | The target population are not women living with HIV |
| 42. | Tong, 2022 | The East Africa consortium for human papillomavirus and cervical cancer in women living with HIV/AIDS | <https://ecommons.aku.edu/cgi/viewcontent.cgi?article=1660&context=eastafrica_fhs_mc_obstet_gynaecol> | No outcome of interest |
| 43. | Tenkir , 2023 | The magnitude of precancerous cervical lesions and its associated factors among women screened for cervical cancer at a referral center in southern Ethiopia, 2021: a cross-sectional study | <https://www.frontiersin.org/journals/global-womenshealth/articles/10.3389/fgwh.2023.1187916/full> | The target population are not women living with HIV |
| 44. | Mutyaba, 2010 | Evaluation of 'see-see and treat' strategy and role of HIV on cervical cancer prevention in Uganda | <https://link.springer.com/article/10.1186/1742-4755-7-4> | No outcome of interest |
| 45. | Derbie, 2022 | Histopathological profile of cervical punch biopsies and risk factors associated with high-grade cervical precancerous lesions and cancer in northwest Ethiopia. | <https://doi.org/10.1371/journal.pone.0274466> | The target population are not women living with HIV |
| 46. | Anna-Lise, 2010 | Polymorphism is associated with increased risk of cervical cancer but not with HPV infection or pre-cancerous lesions in African women | <https://doi.org/10.1186/1471-2407-10-278> | The study was not conducted exclusively in SEA |
| 47. | Klein, 2019 | Relationship between the Cervical Microbiome, HIV Status, and  Precancerous Lesions | <https://doi.org/10.1128/mbio.02785-18> | No outcome of interest |
| 48. | Vuyst, 2012 | Prevalence and determinants of human papillomavirus infection and cervical lesions in HIV-positive women in Kenya | <http://rd3fs2pt9j.search.serialssolutions.com/?ctx_ver=Z39.88-> | No outcome of interest |
| 49. | [Ruche](https://pubmed.ncbi.nlm.nih.gov/?term=La+Ruche+G&cauthor_id=9590121), 1998 | Human papillomavirus and human immunodeficiency virus infections: relation with cervical dysplasia-neoplasia in African women | <https://pubmed.ncbi.nlm.nih.gov/9590121/> | The study was not conducted exclusively in SEA |
| 50. | [Firnhaber](https://pubmed.ncbi.nlm.nih.gov/?term=Firnhaber+C&cauthor_id=19949850), 2010 | Association between cervical dysplasia and human papillomavirus in HIV seropositive women from Johannesburg South Africa | <https://pubmed.ncbi.nlm.nih.gov/19949850/> | No outcome of interest |
| 51. | Suwankanta, 2008 | Underlying histopathology of HIV-infected women with squamous cell abnormalities on cervical cytology | <https://pubmed.ncbi.nlm.nih.gov/18990018/> | The study was not conducted in SEA |
