# Supplementary material for: Precancerous cervical lesions and associated factors among HIV-positive women in Southern and Eastern African countries: A systematic review and meta-analysis
Source: PLoS One. 2026 Oct 23;21(10):e0361185. doi: 10.1371/journal.pone.0361185 (PMC13693988; doi:10.1371/journal.pone.0361185)
Supplement: Supplementary file 5 [file pone.0361185.s006.docx]

**May 26, 2026**

**Rebuttal letter**

**To: PLOS One Editorial Office**

**Authors’ point-by-point Responses to a Manuscript ID: PONE-D-25-63617**

Dear Editor-in-Chief, Greetings.

We have submitted these authors' responses to peer-review comments and questions for a manuscript entitled **"Precancerous cervical lesions and its associated factors among HIV‐positive women in Southern and Eastern African countries: a systematic review and meta-analysis."**

Dear Editor, First, we would like to thank the PLOS One Editorial Office members, especially the chief editor, who timely assigned the competent academic editor and facilitated the progress of the review forum. Second, our special thanks go to the academic editor for his editorial contribution and for assigning the potentially skilled and experienced reviewers in the field promptly. Moreover, our deepest gratitude goes to the esteemed reviewer for his constructive comments and scientific contributions that helped us to improve the quality of this manuscript. Dear Academic Editor, We have made the necessary corrections and responses to those comments raised by the editors and a reviewer point by point, page by page, and line by line sequentially. Accordingly, we have added the authors’ responses in the review forum, as well as the revised manuscript and the tracked changes to the manuscript on the web page based on the instructions to submit the revision.

With kind regards!

Gebrie Getu Alemu

[gebryegetu27@gmail.com](mailto:gebryegetu27@gmail.com)

University of Gondar, Ethiopia

PO. Box 196

Corresponding Author

**Table:** **Authors’ responses to editor and peer-review of reviewers**

| **Academic Editor's comments and Authors’ responses** | |
| --- | --- |
| **Journal Requirements** | **Authors’ Responses** |
| 1 When submitting your revision, we need you to address these additional requirements. Please ensure that your manuscript meets PLOS ONE's style requirements, including those for file naming. The PLOS ONE style templates can be found at  <https://journals.plos.org/plosone/s/file?id=wjVg/PLOSOne_formatting_sample_main_body.pdf>  and <https://journals.plos.org/plosone/s/file?id=ba62/PLOSOne_formatting_sample_title_authors_affiliations.pdf> | Dear editor, thank you for the comment. We have checked the given link to revise the document and we used the author formatting checklist to confirm that our paper meets PLOS ONE's typesetting requirements for references, Tables, and Figures. We ensured that our manuscript meets PLOS ONE’S style including file naming. |
| 2. We note that your Data Availability Statement is currently as follows: All relevant data are within the manuscript and in Supporting Information files. Please confirm at this time whether or not your submission contains all raw data required to replicate the results of your study. Authors must share the “minimal data set” for their submission. PLOS defines the minimal data set to consist of the data required to replicate all study findings reported in the article, as well as related metadata and methods  (<https://journals.plos.org/plosone/s/data-availability#loc-minimal-data-set-definition>).  For example, authors should submit the following data:  - The values behind the means, standard deviations and other measures reported;  - The values used to build graphs;  - The points extracted from images for analysis.  Authors do not need to submit their entire data set if only a portion of the data was used in the reported study.  If your submission does not contain these data, please either upload them as Supporting Information files or deposit them to a stable, public repository and provide us with the relevant URLs, DOIs, or accession numbers. For a list of recommended repositories, please see  <https://journals.plos.org/plosone/s/recommended-repositories>.  If there are ethical or legal restrictions on sharing a de-identified data set, please explain them in detail (e.g., data contain potentially sensitive information, data are owned by a third-party organization, etc.) and who has imposed them (e.g., an ethics committee). Please also provide contact information for a data access committee, ethics committee, or other institutional body to which data requests may be sent. If data are owned by a third party, please indicate how others may request data access. | Dear editor, thank you for the comment. We have no raw data to submit. But all the analyzed data (output) are included in the manuscript in terms of texts, tables and figures. We have submitted the values behind frequencies, median, interquartile range, the values used to build graphs, the points extracted from images for analysis. We said that “All relevant data are within the manuscript and in Supporting Information files” to show the analyzed data and reported in the manuscript. |
| 3. Please include captions for your Supporting Information files at the end of your manuscript, and update any in-text citations to match accordingly. Please see our Supporting Information guidelines for more information: <http://journals.plos.org/plosone/s/supporting-information>. | Dear editor, Thank you for the feedback. We have included captions for Supporting Information files at the end of the manuscript, and we have updated an in-text citations to match accordingly in the revised manuscript. The included captions can be seen on **Page 33,** **Line 724-730.** |
| 4. If the reviewer comments include a recommendation to cite specific previously published works, please review and evaluate these publications to determine whether they are relevant and should be cited. There is no requirement to cite these works unless the editor has indicated otherwise. | Dear editor, Thank you for the comment. As much as possible, we have revised and updated the discussion as per the reviewer recommendation. |
| **Additional editor comments** | **Authors’ Responses** |
| Please note that we have only been able to secure a single reviewer to assess your manuscript. We are issuing a decision on your manuscript at this point to prevent further delays in the evaluation of your manuscript. Please be aware that the editor who handles your revised manuscript might find it necessary to invite additional reviewers to assess this work once the revised manuscript is submitted. | Dear editor, Thank you for the feedback and consideration of the delays. Really, the review process was delayed due to the unavailability of the previously handling editors. If you find it necessary to invite additional reviewers for revision, please feel free to do so. |
| The reviewer's comments can be found below. Could you please revise the manuscript to carefully address the concerns raised? | Dear editor, Thank you for the feedback. We have revised the document according to the reviewer comments. We have worked and addressed the raised comments. We act accordingly based on the comments, feedback, and suggestions raised throughout the manuscript. |
| **Reviewer comments and Authors’ responses** | |
| **Reviewer 1 comments** | **Authors’ Responses** |
| The authors present the results of a meta-analysis of prevalence of cervical precancerous lesions among women living with HIV (WLHIV). This is an important and pertinent topic, especially with the enhancement of survival of WLHIV with HAART. Though the results of the study are interesting, there are a few major concerns, as detailed below: | Dear reviewer, Thank you for picking the important strengths and supporting insight of the research article. We feel a great pleasure for your constructive comments and scientific contributions that helped us to improve the quality of this manuscript. The specific responses for the comments are as follows. |
| 1. The first and foremost concern is the quantitative synthesis of studies using varying cervical cancer screening methods. VIA/ VILI and HPV DNA testing cannot be considered as gold standard for detection of cervical epithelial abnormalities. Studies including cervical cytology only should have been included in the analysis, since only such studies can provide an accurate estimation of the prevalence of cervical lesions in the studied population. Also, authors need to consider that HSIL+ or CIN2+ lesions are considered to be significant cervical lesions for the purpose of cervical cancer screening and control. Hence, even in studies with cervical cytology, the prevalence of such lesion should be quantitatively synthesized or the analysis should be further subgrouped as low-grade lesions and high-grade lesions, considering the widely disparate clinical connotation of these lesions. | Dear reviewer, Thank you very much for the feedback. We have received the comment indicating that it is nice and valuable.  We have reason out why we included articles in the analysis done with VIA/ VILI and HPV DNA testing.  Management algorithms for screen-positive women in cervical cancer prevention programs have undergone substantial changes in recent years. The WHO strongly recommends human papillomavirus (HPV) testing for primary screening, if affordable, or if not, then visual inspection with acetic acid (VIA), and promotes treatment directly following screening through the screen-and-treat approach (one or two clinic visits). **For further information** <https://pubmed.ncbi.nlm.nih.gov/28691336/>  While cytology directly looks at the physical shape and structure of cervical cells to find abnormalities, a primary HPV test checks for the presence of the virus that causes those changes. <https://www.who.int/news-room/fact-sheets/detail/cervical-cancer>  The World Health Organization (WHO) identifies HPV testing as the preferred primary screening method because of its high accuracy and reliability. The **VIA**/**VILI** tests are simple, low-cost, point-of-care screening methods used to detect early, precancerous lesions on the cervix.  <https://cdn.who.int/media/docs/default-source/searo/tobacco/trainees-handbook.pdf?sfvrsn=6778145d_2>  <https://pmc.ncbi.nlm.nih.gov/articles/PMC6457807/>  They are highly recommended by the WHO for cervical cancer screening, particularly in low-resource settings where Pap smear labs are unavailable. VIA is the recommended alternative for settings where HPV testing or cytology (Pap smears) are unavailable or too costly. It is extremely inexpensive, requires minimal equipment, and provides immediate results. If a lesion is found, a healthcare worker can perform immediate treatment on-site. In resource-limited areas, VIA followed by therapy (screen and treat) is an alternative approach to secondary prevention. Since we have conducted this research in low resource settings (Eastern and Southern African countries) most primary studies are conducted using VIA as a screening method. The best screening techniques for cervical cancer in resource-limited settings are VIA/ VILI and HPV DNA testing as recommended by WHO**.** If we include studies screened with cervical cytology (Pap smear) only in the analysis there will be selection bias. Hence the result cannot be generalizable for the population studied in the regions (Eastern and Southern Africa).  Additionally, a leave-one-out sensitivity analysis was employed to assess robustness of findings among the reviewed articles by removing one study at a time. This demonstrated that all the results were contained within the 95% CI, suggesting that no individual study had a substantial influence on the estimated pooled prevalence of precancerous cervical lesions. This verified the reliability of the pooled estimate.  Based on the aforementioned reasons it is better to report the pooled prevalence of precancerous cervical lesion screened with all methods. Unless the finding will be biased, not considering the context, against the WHO recommendations and other guidelines and ultimately not generalizable to the target population in Eastern and Southern Africa.  Regarding the consideration of HSIL+ or CIN2+ lesions, we have quantitatively synthesized their prevalence and included in the revised manuscript. This can be seen **Page 16, Line 289-292.** |
| 2. In view of the above observation, all the subgroup analyses performed in the present study also need to be redone. | Dear reviewer, Thank you for the comment. The shortlisted reasons above (answer for question number 1) are to continue the outcome as it stands. So the subgroup analyses are done aligned with the study objective. |
| 3. The authors should refer to the most recent studies/ analysis of cervical lesions in WLHIV in the Discussion section. | Dear reviewer, Thank you for the comment. The comment is nice and constrictive. As much as possible, we have revised and updated the discussion as per the recommendation. We have revised and cited recent sources (references in 2020 and later). This can be seen in the discussion section of the revised manuscript. |
| 4. Why was the analysis confined to studies published from 2015? Cervical cancer screening methods were in use prior to this year also. The authors may clarify this. | Dear reviewer, Thank you for the comment. We have included studies published in 2015 and later for the following reasons.  1. In January 2015, the American Society for Colposcopy and Cervical Pathology and the Society of Gynecologic Oncology issued **clinical guidance.** This was a direct response approval of HPV test for primary screening. For the first time, using an HPV test alone was considered an acceptable option for cervical cancer screening in women.  2. Later in 2015, the American College of Obstetricians acknowledged the interim guidance, stating that primary HPV testing alone could be considered an alternative to cytology for women.  3. On **March 19, 2015**, the German Federal Joint decided to introduce an organized cervical cancer screening program. This decision officially implemented the HPV test as the primary screening tool for women aged 30 and older, moving away from the Pap smear as the first-line test.  4. To synthesize the recent evidence (data within 10 years) which is important for policy recommendations.  For the aforementioned reasons we have included studies published in 2015 and later. |
| 5. Subgroup analysis of studies published before and after 2020 has been shown to demonstrate a difference in prevalence. Does this reflect the true prevalence or the effect of COVID pandemic on the utilization of cervical cancer screening services? | Dear reviewer, Thank you for the important insight. Really, the difference could be due to the effect of COVID 19 pandemic. The COVID 19 pandemic severely disrupted cervical cancer screening in resource-limited settings in addition to the existing challenges. We have tried to justify the reason as follows.   1. COVID-19 caused a severe decline in cervical cancer screenings globally due to clinic closures, diverted healthcare resources, and patient reluctance to seek care. This massive drop in Pap smears and HPV tests delayed the detection of pre-cancerous lesions, ultimately increasing the risk of advanced cervical cancer diagnoses and mortality. Consequently, the prevalence of PCCL decreased. 2. Cervical cancer screening dropped dramatically during the pandemic's peak, particularly in early 2020. During the peak of the pandemic, routine cervical smear submissions and screenings dropped significantly (with declines up to 30-40% in some regions) as healthcare resources shifted and clinics closed. 3. Non-adherence to screening due to COVID-19 pandemic disproportionately affected under-represented populations (WLHIV) in lower socioeconomic groups (Southern and Eastern Africa).   Due to these reasons the prevalence of precancerous cervical lesions decreased during the COVID-19 pandemic when compared with the prevalence of PCCL before 2020.  We have justified the difference in the prevalence of PCCL before 2020 and later with respect the effect of COVID-19. This can be seen **Page16-17, Line 308-313.** |
