# Supplementary material for: Precancerous cervical lesions and associated factors among HIV-positive women in Southern and Eastern African countries: A systematic review and meta-analysis
Source: PLoS One. 2026 Oct 23;21(10):e0361185. doi: 10.1371/journal.pone.0361185 (PMC13693988; doi:10.1371/journal.pone.0361185)
Supplement: Supplementary file 6 [file pone.0361185.s007.docx]

**September 15, 2026**

**Rebuttal letter**

**To: PLOS One Editorial Office**

**Authors’ point-by-point Responses to a Manuscript ID:** PONE-D-25-63617R1

Dear Editor-in-Chief, Greetings.

We have submitted these authors' responses to peer-review comments and questions for a manuscript entitled **"Precancerous cervical lesions and its associated factors among HIV‐positive women in Southern and Eastern African countries: a systematic review and meta-analysis."**

Dear Editor, First, we would like to thank the PLOS One Editorial Office members, especially the chief editor, who timely assigned the competent academic editor and facilitated the progress of the review forum. Second, our special thanks go to the academic editor for his editorial contribution and for assigning the potentially skilled and experienced reviewers in the field promptly. Moreover, our deepest gratitude goes to the esteemed reviewer for his constructive comments and scientific contributions that helped us to improve the quality of this manuscript. Dear Academic Editor, We have made the necessary corrections and responses to those comments raised by the editors and a reviewer point by point, page by page, and line by line sequentially. Accordingly, we have added the authors’ responses in the review forum, as well as the revised manuscript and the tracked changes to the manuscript on the web page based on the instructions to submit the revision.

With kind regards!

Gebrie Getu Alemu

[gebryegetu27@gmail.com](mailto:gebryegetu27@gmail.com)

University of Gondar, Ethiopia

PO. Box 196

Corresponding Author

**Table:** **Authors’ responses to editor and peer-review of reviewers**

| **Journal Requirements** | |
| --- | --- |
|  |  |
| **Journal Requirements** | **Authors’ Responses** |
| 1. If the reviewer comments include a recommendation to cite specific previously published works, please review and evaluate these publications to determine whether they are relevant and should be cited. There is no requirement to cite these works unless the editor has indicated otherwise. | Dear editor, Thank you for the comment. As much as possible, we have revised and updated the most important and current references in the revised document. |
| **Editor comments** | **Authors’ Responses** |
| As you can see, the reviewers have requested substantial revisions to your manuscript, including language editing. We are certainly willing to reconsider a revised submission, but please know that this is not preliminary acceptance of your paper. When returning your revised manuscript, please be sure to include a point-by-point summary of the suggestions of the reviewers that specifies how and where in the text you have addressed the suggestions. | Dear editor, Thank you very much for your email and for the opportunity to revise our manuscript. We understand that the reviewer has requested substantial revisions, including language editing. We also note the requirement to include a point-by-point summary of the reviewers' suggestions that specifies how and where in the text each suggestion has been addressed. We have carefully considered all of the reviewers' comments and prepared a revised manuscript accordingly. Along with the revised submission, we have provided a detailed point-by-point response document that outlines each suggestion and indicates the corresponding changes and their locations in the revised text. We have also ensured that the language is thoroughly edited before resubmission. We have used language editing services. |
| **Reviewer comments and Authors’ responses** | |
| **Reviewer 1 comments** | **Authors’ Responses** |
|  | **Dear reviewer, we feel a great pleasure for your constructive comments and scientific contributions that helped us to improve the quality of this manuscript. We have tried to address the comments and suggestions. The specific responses for the comments are as follows.** |
| 1. Though I agree that cervical cancer screening is advocated to be done by the feasible and available modality, the authors should attempt to do a subgroup analysis of studies segregated by the screening modality utilized. This is important since the prevalence of precancerous lesions would vary by the modality used. | Dear reviewer, Thank you very much for the feedback. We have received the comment indicating that it is nice and valuable.  We have conducted a subgroup analysis by the screening methods. Really, the prevalence of precancerous cervical lesions varies by the screening methods.  The subgroup analysis can be seen in **Figure 5, and the textual explanations in page 18-19, Line 319-337.** |
| 2. I still do not concur with the reason of limiting the search to post-2015 just on the basis of ASCCP guidelines of using HPV-based assays for primary cervical cancer screening. Screening by VIA or cytology or even HPV has been going on much before that. Restricting the search to post-2015 is likely to bias the results, since more studies on HPV screening may be included, thereby skewing the results. | Dear reviewer, Thank you for your thoughtful and critical review of our methodology. You raise a very valid point regarding the potential for selection bias by limiting the search to post-2015, and we appreciate the opportunity to clarify our rationale. We fully agree that searching from inception is the gold standard for avoiding publication bias. We want to reassure you that we **did** perform an inception search (without date restriction).As a result, we have additionally included 11 articles. We have revised the whole parts of the document including the supplementary materials and search strategy. We ensure you that the generalizability of the document is based on the included studies without date restriction and we believe that selection bias resulted from date restriction is avoided. **The revision can be seen in the current submitted manuscript starting from the abstract.** **Kindly see the revision.** |
| 3. "The sub-group analysis of PCCL by geographical region was high among the studies conducted in Southern Africa which was 25.91% ". Reframe sentences like this. | Dear reviewer, Thank you for the constrictive and invaluable feedback. The comment is nice and constrictive. We have revised and updated the sentence as per the recommendation. This can be seen **Page 17, Lines 297-299**. As much as possible, we have refined other sentences as per the given direction. |
| 4. The subgroup analysis of before and after 2020 is fine. If the effect of COVID-19 pandemic is to be considered, it might be prudent to do an analysis from 2020 till 2022 and 2023-2025 to see if the lower prevalence was really due to the pandemic and corrected itself once the pandemic had passed. | Dear reviewer, Thank you for this interesting and constructive suggestion. We are grateful for this suggestion You are right while comparing pre-2020 and post-2020 gives a gross estimate of the pandemic's impact. It is more elegant and intellectually honest to divide the post-2020 timeframe into two phases: 2020–2022 (acute pandemic/disruption phase) and 2023–2025 (post-pandemic stabilizing phase). So we have conducted a subgroup analysis by dividing the time frame into four. Pre 2015, 2015-2019, 2020-2022, and post 2022. Really in the subgroup analysis the lowest overall prevalence of precancerous cervical lesions occurred during the year 2020-2022. We have justified the lowest prevalence of PCCL from 2020-2022 is due to the effect of COVID-19 pandemic in the revised document. **The subgroup analysis can be seen in Figure 4, Page 18, Line 307-318, in the revised manuscript. Kindly see the revision.** |
| 5. The subgroup analysis by sample size seems scientifically incorrect. The prevalence is affected more by factors such as duration of HIV infection, adherence to HAART therapy, and sexual practices more than the sample size. Hence, this confusing and somewhat incorrect analysis should be removed. | Dear reviewer, Thank you for the important insight. We are grateful for your careful reading and your willingness to increase the quality of the paper. We have removed the subgroup analysis previously done by the sample size. The sample-size subgroup analysis entirely from the results section, including all corresponding forest plots and textual explanations are removed in the revised manuscript. |
| 6. For the quantitative synthesis of factors associated with precancerous cervical lesions in women living with HIV, it is imperative that the studies being included for each individual factor is matched with respect to many factors. As is evident from the forest plots, there is a high heterogeneity in majority of the associated factors. At the same time, the studies should be using the same screening modality since the prevalence itself changes with the modality and the associated factors would differ as well. Hence, the authors need to look into this analysis in depth again and discuss with the statistician for the same.  We sincerely thank the reviewer for this insightful and important observation regarding the quantitative synthesis of factors associated with precancerous cervical lesions in women living with HIV. | Dear reviewer, we sincerely thank for this insightful and important observation regarding the quantitative synthesis of factors associated with precancerous cervical lesions in women living with HIV. We fully acknowledge the concerns raised. Really one important challenge when pooling data from several primary studies is heterogeneity which is un avoidable problem, but it can be explainable.  We have consulted with the statistician to ensure that the analytical approach is appropriate and that any revised analyses are methodologically sound. Due to the heterogeneity, we have used **a random-effects model** to account for variability among the studies, which assumes multiple true effect sizes. Since the random-effects model was used for statistical analysis, the interpretability of the pooled results cannot be limited. The random-effects model gives more balanced and generalized estimates across variable studies.  Most heterogeneities for the factors are reduced on reanalysis by including articles before 2015. We have removed factors which have high heterogeneity (I² > 75%). For overall of the heterogeneity we have briefly acknowledged as a limitation, and the possible influence on the pooled estimate has been discussed. **Kindly see the revisions in Fig 8, 9 and the textual explanations from page 20-22, Lines 366-410.** |
