# Supplementary material for: Precancerous cervical lesions and associated factors among HIV-positive women in Southern and Eastern African countries: A systematic review and meta-analysis
Source: PLoS One. 2026 Oct 23;21(10):e0361185. doi: 10.1371/journal.pone.0361185 (PMC13693988; doi:10.1371/journal.pone.0361185)
Supplement: Supplementary file 7 [file pone.0361185.s008.docx]

**September 30, 2026**

**Rebuttal letter**

**To: PLOS One Editorial Office**

**Authors’ point-by-point Responses to a Manuscript ID:** PONE-D-25-63617R2

Dear Editor-in-Chief, Greetings.

We have submitted these authors' responses to peer-review comments and questions for a manuscript entitled **"Precancerous cervical lesions and associated factors among HIV‐positive women in Southern and Eastern African countries: a systematic review and meta-analysis."**

Dear Editor, First, we would like to thank the PLOS One Editorial Office members, especially the chief editor, who timely assigned the competent academic editor and facilitated the progress of the review forum. Second, our special thanks go to the academic editor for his editorial contribution and for assigning the potentially skilled and experienced reviewers in the field promptly. Moreover, our deepest gratitude goes to the esteemed reviewer for his constructive comments and scientific contributions that helped us to improve the quality of this manuscript. Dear Academic Editor, We have made the necessary corrections and responses to those comments raised by the editors and a reviewer point by point, page by page, and line by line sequentially. Accordingly, we have added the authors’ responses in the review forum, as well as the revised manuscript and the tracked changes to the manuscript on the web page based on the instructions to submit the revision.

With kind regards!

Gebrie Getu Alemu

[gebryegetu27@gmail.com](mailto:gebryegetu27@gmail.com)

University of Gondar, Ethiopia

PO. Box 196

Corresponding Author

**Table:** **Authors’ responses to editor and peer-review of reviewers**

| **Journal Requirements** | |
| --- | --- |
|  |  |
| **Journal Requirements** | **Authors’ Responses** |
| 1. If the reviewer comments include a recommendation to cite specific previously published works, please review and evaluate these publications to determine whether they are relevant and should be cited. There is no requirement to cite these works unless the editor has indicated otherwise. | Dear editor, Thank you for the comment. As much as possible, we have revised and updated the most important and current references in the revised document. |
| **Editor comments** | **Authors’ Responses** |
| As you can see, the reviewers have requested substantial revisions to your manuscript; there is an urgent requirement of language editing and improvement of the structure of the manuscript. We are certainly willing to reconsider a revised submission, but please know that this is not preliminary acceptance of your paper. When returning your revised manuscript, please be sure to include a point-by-point summary of the suggestions of the reviewers that specifies how and where in the text you have addressed the suggestions. | Dear editor, Thank you very much for your email and for the opportunity to revise our manuscript. We appreciate the reviewer's comments which has improved our manuscript's methodological rigor and contextual framing. We understand that the reviewer has requested substantial revisions, including language editing. We also note the requirement to include a point-by-point summary of the reviewers' suggestions that specifies how and where in the text each suggestion has been addressed. We have carefully considered all of the reviewers' comments and prepared a revised manuscript accordingly. Along with the revised submission, we have provided a detailed point-by-point response document that outlines each suggestion and indicates the corresponding changes and their locations in the revised text. We have applied the Grammarly tool to improve the language of the manuscript and we have revised by discussing with the authors. We have tried our best to improve the readability of the manuscript and correct grammatical errors. |
| **Reviewer comments and Authors’ responses** | |
| **Reviewer 1 comments** | **Authors’ Responses** |
| 1. Introduction section is too long and contains unnecessary details such as the three modalities of cervical cancer screening that are well known. The section begins with an abbreviation (PCCL) that should be expanded since this is the first occurrence in the main manuscript. | Dear reviewer, thank you for the comment. We have found the comment is constructive and invaluable. We have tried to shorten the introduction sections of the manuscript without compromising the message by reducing the over lengthy paragraphs. We have removed the unnecessary wordings and sentences; we try to make the introduction catchy with the important information. This can be seen **Page 3-5, line 68-107** in the revised manuscript.  We have first mention the full texts of PCCL and the abbreviation in bracket. This can be seen **Page 3, line 69.** We have used other abbreviations by defining them first clearly in their full texts and then used the abbreviations consistently throughout the manuscript. |
| 2. The protocol registered on PROSPERO has not been updated with respect to the change in search timelines, methodology of review, etc. | Dear reviewer, thank you for the important insight. Really we have missed to update the registered protocol in the PROSPERO on the second revision and submission. But now we have updated the PROSPERO with respect to the timeframe for the included studies, methodology and others in line with the current manuscript.  **Kindly see the link given here for the updated PROSPERO**. <https://www.crd.york.ac.uk/PROSPERO/view/CRD420251123583> |
| 3. The Eligibility criteria are incomplete. These need to be written in a complete and concise manner. The authors are advised to refer to some recently published meta-analysis on a similar topic for guidance. "Studies were excluded if they did not present the outcome of interest, qualitative reviews, case studies, letters, protocols, methodological reviews, and articles that failed to satisfy the quality assessment standards." What was the cut-off used to satisfy quality assessment standards? | Dear reviewer, Thank you for the constrictive and invaluable feedback. We appreciate the comment. We have revised and updated the eligibility criteria to be complete, concise and clear. This can be seen **Page 5-6, Lines 113-119**. We have referred and cited recently published related articles for the exclusion. This can be seen **Page 6, Lines 117-119.**  Regarding to the question “**What was the cut-off used to satisfy quality assessment standards?”** We have typically used the published documents to determine scoring and cutoff scores for including or excluding studies based on the Newcastle-Ottawa Scale (NOS) in systematic reviews. These documents state that:  Very good quality: 9–10 points; studies in this range are generally included without reservations.  Good quality: 7–8 points; studies are usually included as they meet acceptable methodological standards.  Satisfactory quality: 5–6 points; fall into the satisfactory category, showing moderate quality but with some limitation and to be included.  Unsatisfactory quality: 0–4 points; studies are commonly excluded due to high risk of bias or poor methodological quality.  Many systematic reviews adopt the above NOS thresholds as practical standards for inclusion/exclusion decisions. We have already summarized this on **Page 7, Lines 152-161, and Supplementary Table 3 (S3 Table).**  We have cited some of the sources in which scholars adapt and used for quality assessment of studies in the revised manuscript **Page 7, line 161** and the links here.  <https://bmjopenrespres.bmj.com/content/bmjresp/10/1/e002076/DC2/embed/inline-supplementary-material-2.pdf?download=true>  <https://repository.up.ac.za/server/api/core/bitstreams/4bae9e5e-b9a0-4216-8525-71f553c0f15a/content> |
| 4. "All selected studies were imported into EndNote 20 for removal". What does this mean? The manuscript is full of numerous such statements that need to be looked at thoroughly by the authors. The authors may take assistance from a language editing tool, if required. | Dear reviewer, thank you for pointing this. We agree that the original sentence was unclear and methodologically imprecise. Our intention was to state that all records retrieved from the database searches were imported into EndNote 20, and duplicate records were removed before screening. We have revised the sentence as follows: “All retrieved records were exported into EndNote 20 to remove duplicates and then evaluated for eligibility”. We have also carefully reviewed the entire manuscript for similar unclear or vague statements. Terminology and phrasing have been corrected throughout to improve clarity, consistency, and methodological accuracy. The revised manuscript has been checked for language and clarity with the Grammar tool and discussion within the authors and all changes are marked in the tracked-changes version. |
| 5. The subsection on 'Identification and selection of the studies' may be shortened. Since the PRISMA flowchart has been provided, all the details may not be replicated in text. Only salient numbers may be provided in text. | Dear reviewer, Thank you for the important insight. We are grateful for your careful reading and we have shortened and modified as per the recommendation. The revisions can be seen **Page 9-10, Lines 206-212.** |
| 6. "The subgroup analysis of PCCL by geographical region was high among the studies conducted in Southern Africa, which was 31.08%". Statements like this need to be reframed. | Dear reviewer, we sincerely thank for this insightful and important observation regarding the statement clarity. We fully acknowledge the concerns raised. The comment is nice and constrictive. We have revised and updated the sentence as per the recommendation. **This can be seen Page 14, Lines 247-248.** As much as possible, we have refined other sentences as per the given direction. |
| 7. The results section is intermixed with discussion. The authors need to be careful about this. | Dear reviewer, thank you for the comment. We acknowledge that our original manuscript intermixed result elements into the discussion section and vice versa and we have now carefully revised the manuscript to separate these two sections completely. In the result section we have described the output of the data analysis that are relevant to the study objectives. Really, we have mixed the discussion part in the interpretation section for the factors associated with PCCL in the result and the section have been updated. This can be seen **Page 17-19, Lines 313-350.**  In the discussion we have removed the result sections in which their presence is irrelevant. But, we re-stated the main findings especially for the primary outcome of interest for interpretations, opinions and to explain the implications of the findings. So, we highlighted the finding to compare others finding whether their figure was in our confidence interval or not. We have also highlighted important information for the factors. This makes the reader easily understand the interpretation. So, we have re-stated the main findings and interpret the result in context of literatures. Kindly see the results and discussion in the revised manuscript. |
| 8. The subgroup analysis by year of publication should also mention the number of women screened during the duration included to emphasize the decline in numbers due to the pandemic. | Dear reviewer, thank you for the important feedback. You are right while mentioning the number of women screened during the duration gives a more elegant and intellectually honest estimate. So, we have mentioned the sample size for the screening duration: As before 2019 (before the COVID-19 pandemic), from 2020 to 2022 (during the COVID-19 pandemic), and post 2022 (after the COVID-19 pandemic). The number of women screened before 2019, from 2020 to 2022, and post 2022 were 12052, 7668, and 27289, respectively. Really, in the subgroup analysis, the lowest overall prevalence of precancerous cervical lesions and the lowest number of women screened occurred from 2020 to 2022 (during the COVID-19 pandemic). We have included the number of women screened during the duration in the revised figure.  We have justified the lowest prevalence of PCCL from 2020 to 2022 as due to the effect of the COVID-19 pandemic. The subgroup analysis can be seen in **Figure 4, and in the manuscript on Page 15, Lines 256-267. Kindly see the revision.** |
| 9. "It is important to note that the majority of earlier studies included both HIV-positive and HIV-negative women". Please give references. | Dear reviewer, thank you for the feedback. We have cited the appropriate references. This can be seen **Page 19, Line 361.** |
| 10. "Factors such as compromised immunity and a higher risk of persistent HPV infection make WLHIV more susceptible to PCCL.” Please give references. | Dear reviewer, thank you for suggestion. We have cited the appropriate references as per the recommendation. This can be seen **Page 19, Line 364.** |
| 11. Majority of the statements in the Discussion section do not have references. | Dear reviewer, thank you for the important and invaluable observation. We acknowledge that the majority of statements in the discussion section lacked supporting references, and we have now comprehensively revised the section to address this. In order to contextualize our findings within the body of current literature, we have added citations to all substantial statements. There are some statements without citation. These are our (the authors) idea. We have used these to indicate the **implications** of the findings for policy and practice which is part of the discussion. |
| 12. The manuscript requires stringent language editing and reorientation of sections. The readability of the manuscript in its present form is very low. | We sincerely thank you for this invaluable and constructive assessment. We fully accept that the manuscript in its previous form suffered from poor readability and suboptimal section organization, and we have taken substantial steps to address these issues comprehensively. We have applied the Grammarly tool to improve the language of the manuscript and we have revised by discussing with the authors. We have tried to improve the readability of the manuscript and correct grammatical errors. We have try our best to write our manuscript based on the art of clear communication "3 C's” (Conciseness, Completeness and Clarity). |
